# A fish-friendly axial flow pump turns out to be eel safe, roach unfriendly and bream unsafe

**DOI:** 10.1038/s41598-024-81095-6

**Published:** 2024-12-04

**Authors:** Stijn Bruneel, Ine S. Pauwels, Sarah Broos, Lore Vandamme, Jeroen Van Wichelen, Johan Coeck, Gert Toming, Jeffrey A. Tuhtan, David Buysse

**Affiliations:** 1 Research Institute for Nature and Forest (INBO), Havenlaan 88 bus 73, 1000 Brussels, Belgium; 2 Department of Computer Systems, Tallinn University of Technology, Tallinn, Estonia; 3 Hydraulics Laboratory, Department of Civil Engineering, Ghent University, Ghent, Belgium

**Keywords:** Fish-friendly Fairbanks Nijhuis axial flow pump, Fish passage, Barotrauma detection system sensors, Biological techniques, Civil engineering, Behavioural ecology, Freshwater ecology, Animal migration

## Abstract

Additional and refurbished pumping stations are required to mitigate the intensifying occurrence of droughts and floodings. These installations negatively impact threatened freshwater fish populations due to the increased risk of injury and mortality when fish pass through them. Fish-friendly pumping installations have been proposed as a potential solution to reduce these risks. However, published assessments of these new types of pumps remains lacking, and the few available studies do not enable a cross-comparison with conventional pump types. The promising, yet understudied, Fairbank Nijhuis ‘fish-friendly’ axial flow pump has been assessed in previous works, however the results remain ambiguous due to low recapture rates, unconsidered parameters, fixed operating conditions, and the inability to identify the likely sources of injury and mortality. In this study, we address the limitations of previous works by implementing a standardized protocol for live fish in conjunction with passive barotrauma detection sensors. The major finding of this work is that safe passage of eel (100% survival) is confirmed, but that bream and roach had a much lower survival probability (24% and 70% survival respectively) than expected, albeit higher than for a conventional axial flow pump (roach survival: 13%). Furthermore, roach and bream passing at higher rpm suffered significantly higher mortalities. The impact of the impeller was found to be the most common source of severe injury for both pumps. These results are significant because they conclusively show that fish-friendly pumps may be considered safe for eel, but not for other endemic European fish species such as roach and bream.

## Introduction

Axial flow pumps or screw pumps are used worldwide to transport water for flood control, irrigation, and water provisioning. They are the preferred option when dealing with high flow rates and low vertical distances (e.g.< 5 m) over which water must be transported^1^. In lowland regions prone to flooding, such as Flanders^2^ and the Netherlands^3^, they remain the most widely used pumps in canals and rivers. Unfortunately, they are also among the most damaging for passing fish due to their high specific speed and relatively small size^1,4,5^. Although the European regulatory community is well aware of the threats that barriers in rivers pose to aquatic ecosystems^6^ and intends to reduce their number^7^, it is unlikely that barriers like pumping stations will be removed any time soon. On the contrary, the increase in salinization^8^, the occurrence of droughts^9^ and the intensity of heavy precipitation events^10^, associated with climate change^11^, increase the need for pumping installations^12,13^.

As pumping installations will remain a constant threat to migrating fish^14^, engineers are tasked with designing fish safe or at a minimum, fish-friendly configurations^1,15,16^. Although there have been many studies on the fish-friendliness of turbines, which operate in a reverse way (i.e. producing electricity from the use of falling or fast-running water also known as hydropower), pumps have received considerably less attention. The use of hydropower as renewable energy source is ambiguous given the threat it poses to aquatic ecosystems^17,18^. Furthermore, the efficiency of hydropower installations is expected to suffer drastically under a changing climate^19^. Despite these weaknesses and threats, hydropower is most likely to become an even more important source of energy in the future^20,21^, justifying more research to mitigate its effects as much as possible. At least a similar increase in research is needed for pumping installations as it will face many of the same future challenges as turbines. Unlike hydropower however, for which multiple alternatives exist^17^, there are no real engineering alternatives to deal with the required water management purposes that pumping installations are used for^22^.

Pumps can be made safer by reducing the number of collisions, pressure variation, turbulence, shear stress and cavitation forces. Since the severity and the source of the injuries fish endure seems to depend on the species (and associated behavior), body size, pump type, and pump operation, the conditions, design, adaptation and use of pump installations need to be well-considered. The Fairbanks Nijhuis’ patented “fish-friendly” axial flow pump (FNAFP) claims to accommodate the safe passage of 100% of all eel and at least 97% of all other fish^23^. However, these results are based on a rather small study, with fixed operating conditions and questionable sampling design and associated assumptions regarding the source of the majority of the injuries. A more recent study by^24^ paints another picture as mortality in the FNAFP seems to be as high, and for some species even higher, as for conventional pumps. Though a more standardized injury protocol was used^25^, the study still suffers from many of the aforementioned shortcomings such as a small sample size.

There have been some studies that aimed to simulate the conditions within fish-friendly pump installations in order to assess their safety^1,26^. Although sensors nowadays provide some level of validation of these simulations, the link with live fish remains hypothetical. The few in-situ assessments of pump installations that make use of live fish are often under-powered and do not consider fish length^23,24^. Especially for the FNAFP, the contradicting results of the aforementioned studies in combination with the observed limitations within, warrant a new assessment to serve as baseline. We argue that live fish, a standardized injury protocol, fish length measurements and sensors are needed to obtain an accurate estimate of fish-friendliness and to determine the source of the injuries. In this study on the fish-friendliness of the FNAFP we ensured to have at least 100 individual fish per scenario forced through the FNAFP (bream: > 100 individuals, eel: > 250 individuals, roach: > 300 individuals), employed a standardized injury protocol, and considered both the fish length and the species as potentially influential factors. Furthermore, we provided a more fundamental assessment of the injury sources of the pump via barotrauma detection sensors (BDS). Finally, the data of the forced experiment was compared with data of natural passage via a conventional axial flow pump (CAFP; for which forced tests are strongly discouraged from an ethical perspective) at the same site.

## Results

### Fish data analysis

#### Recapture rate

Although the overall recapture rate (i.e the total number of captured over the total number of released fish for all scenarios) was high for bream (99.34%, 300 of 302 individuals), roach (86.57%, 909 of 1050 individuals) and eel (88.14%, 617 of 700 individuals), there was substantial variation in the recapture rate per scenario (ranging from 63 to 210%: Table 2) due to differences between the theoretical and observed scenarios: fish intended for a certain scenario (i.e. theoretical scenario) sometimes ended up in another subsequent scenario (i.e. observed scenario) as they lingered before the entrance of the installation and only went through after another scenario had already started. It was not possible to ascertain whether fish lingered, otherwise they would have been removed before the start of a new scenario. This was most pronounced for bream, for which fish intended for the low rpm (468 rpm) regime scenario ended up in the subsequent high rpm (550 rpm) regime scenario. This caused the recapture rate of the low and high rpm scenarios to be lower and higher than 100% respectively.

#### Fish length

Average fish length was not significantly different between scenarios for roach (ANOVA, *p* = 0.51) and eel (ANOVA, *p* = 0.56), but was marginally significantly different (if a p-value is between 0.05 and 0.10 it is further on referred to as marginally significant) between scenarios for bream (ANOVA, *p* = 0.086.) (Supplementary Fig. S1).

#### Fish survival

**Fig. 1:**
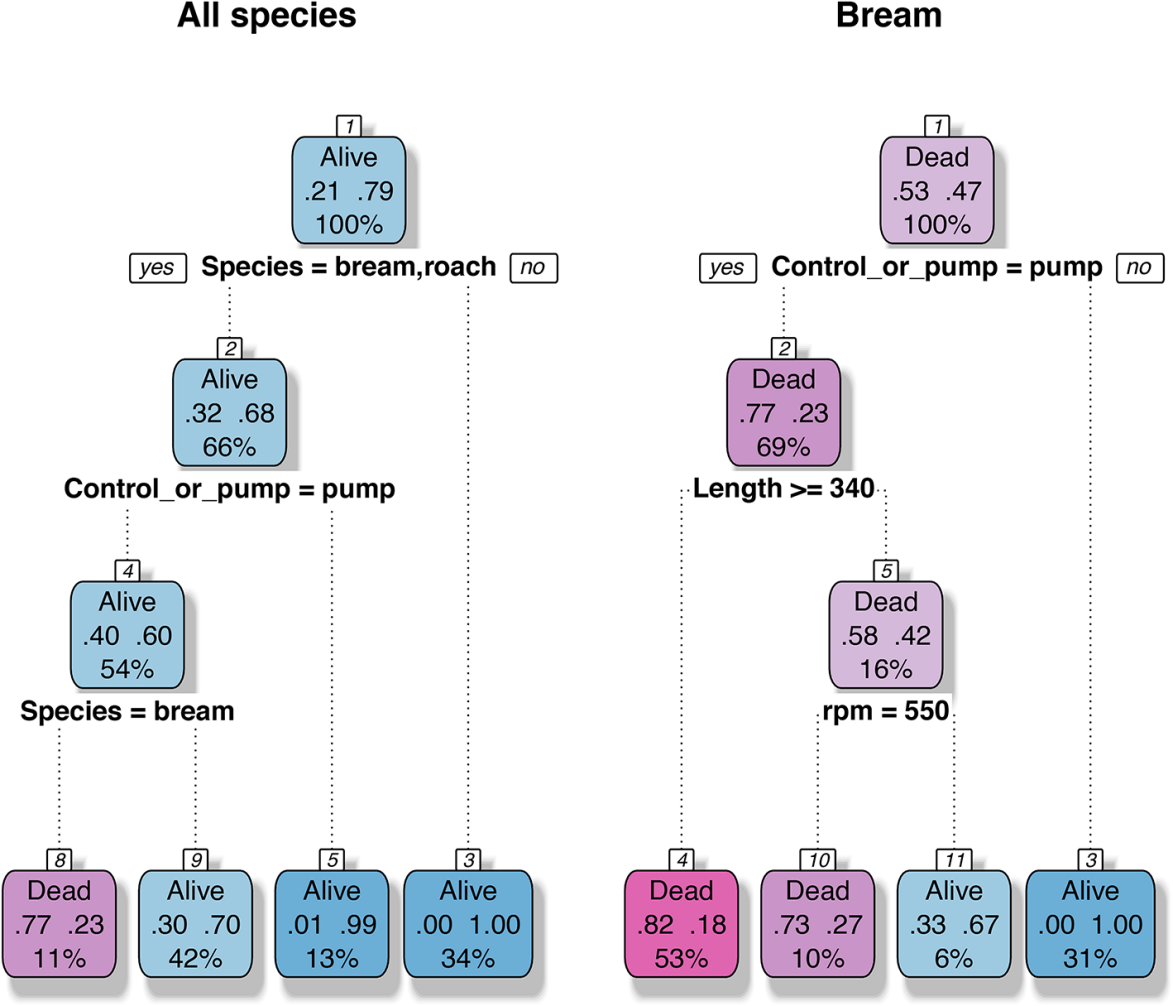
Decision trees for the FNAFP with survival (survived or died) as binary response. For the decision tree constructed with all data (i.e. all species, left decision tree), the factors species, type (pump or control), rpm and fish length (in mm) were provided. For the decision tree constructed with the bream data (right decision tree), the factors type (pump or control), fish length and rpm were provided. The first two numbers next to each other in each branch give the proportion of dead (left) and alive (right) fish. The third number at the bottom gives the percentage of fish the node applies to. Nodes are labeled as Dead or Alive when the proportion of dead fish is higher or lower than 50% respectively. Nodes are numbered in the following way: the main branch at the top receives the number one and from there on, the numbers increase from left-to-right and downwards. The first branch under the main branch to the left will receive number two and the second branch under the main branch to the right will receive the number three, and so on. Some numbers are skipped because non-existing nodes are also numbered. Left branches always represent an affirmation to the question at each parting of branches. The color range from pink to blue indicate whether fish were more likely to be dead or alive. *FNAFP* Fairbanks Nijhuis Axial Flow Pump.

When considering the FNAFP decision tree for all three species it became clear that the species and type of sample (i.e. control versus pump-passage) were most important to estimate the survival rate (Fig. 1). Eel (either belonging to the control or pump-passage samples) had a survival rate of 100%. The remaining control samples, consisting entirely of roach and bream, had a survival rate of 99% (which can be traced back to one roach with a severe injury). The last node of the decision tree distinguished between bream (23% survival rate) and roach (70% survival rate). Since all roach were smaller than all bream, this criterium could also have been perfectly replaced by a node that distinguishes between fish larger or smaller than 200 mm. It is therefore difficult to disentangle whether the higher survival probability of roach compared to bream is because of (i) the lower length of the former (population average of 168 mm for roach versus a population average of 374 mm for bream), (ii) the species-specific behaviour and/or vulnerability, or (iii) a combination of both. When considering the species separately, no decision tree could be developed for roach or eel, because the factors (pump passage versus control, rpm and fish length) had no strong effect on their observed survival rate. For bream an obvious first distinction was made based on whether a fish passed the pump (23% survival rate for the pump passage samples) or not (100% survival rate for the control samples) (Fig. 1). Bream that were smaller than 340 mm had a 67 and 27% survival chance if they went through the pump when the rpm was low versus high respectively. Bream larger than 340 mm had a survival rate of 18%, regardless of the rpm regime.

**Fig. 2:**
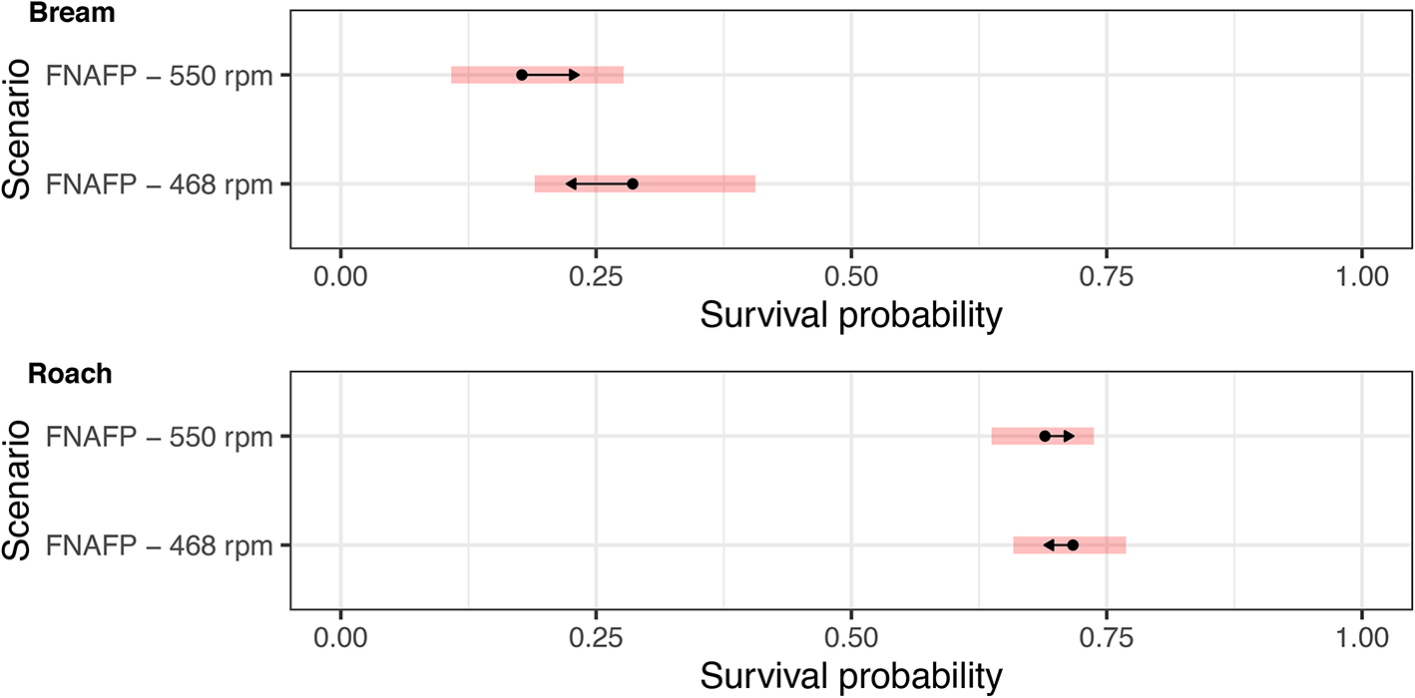
Model output of the most parsimonious logistic survival models for bream (for a fish with an average length of 374 mm) and roach (for a fish with an average length of 168 mm). 95% confidence intervals are given per species and scenario as red bands. Non-overlapping arrows indicate a significant difference (*p* < 0.05). *FNAFP* Fairbanks Nijhuis Axial Flow Pump.

The logistic models indicated that survival of bream was lower at higher rotations with marginal significance (*p* = 0.077) (Fig. 2 and Supplementary Table S1). At lower rotations, larger bream had a significantly (*p* = 0.005) lower chance of survival (Supplementary Fig. S3 and Supplementary Table S1). At higher rotations the much weaker negative effect of length on survival chance was non-significant (*p* = 0.3545). Specifically, at low rpm the survival rate for smaller bream (< 300 mm) was approximately 50% higher than at high rpm, but the difference in survival rate between both rpm regimes became almost zero for the largest fish (> 400 mm; Supplementary Fig. S3). Similarly, survival of roach was lower at higher rotations with marginal significance (*p* = 0.096). At lower rotations, larger roach had a significantly lower chance of survival (*p* = 0.010). At higher rotations the much weaker negative effect of length on survival chance was non-significant (*p* = 0.71). The difference in survival rate between fish of different lengths is therefore also bigger for roach at low rpm than at high rpm. Unlike bream however, the difference for larger fish between the two rpm scenarios does not become negligible, but rather indicates a lower survival rate for the largest fish at low (468 rpm) rpm compared to high (550 rpm) rpm. This is an unexpected result, yet it does seem to represent the data accurately and does not seem to be the result of some remaining outliers. When fish length was not accounted for in the model, the effect of rpm scenario remained marginally significant (*p* = 0.08) for bream, but became non-significant (*p* = 0.45) for roach. Neither rpm nor length had a any observable effect on the survival of eel as all eels survived in both scenarios.

**Fig. 3:**
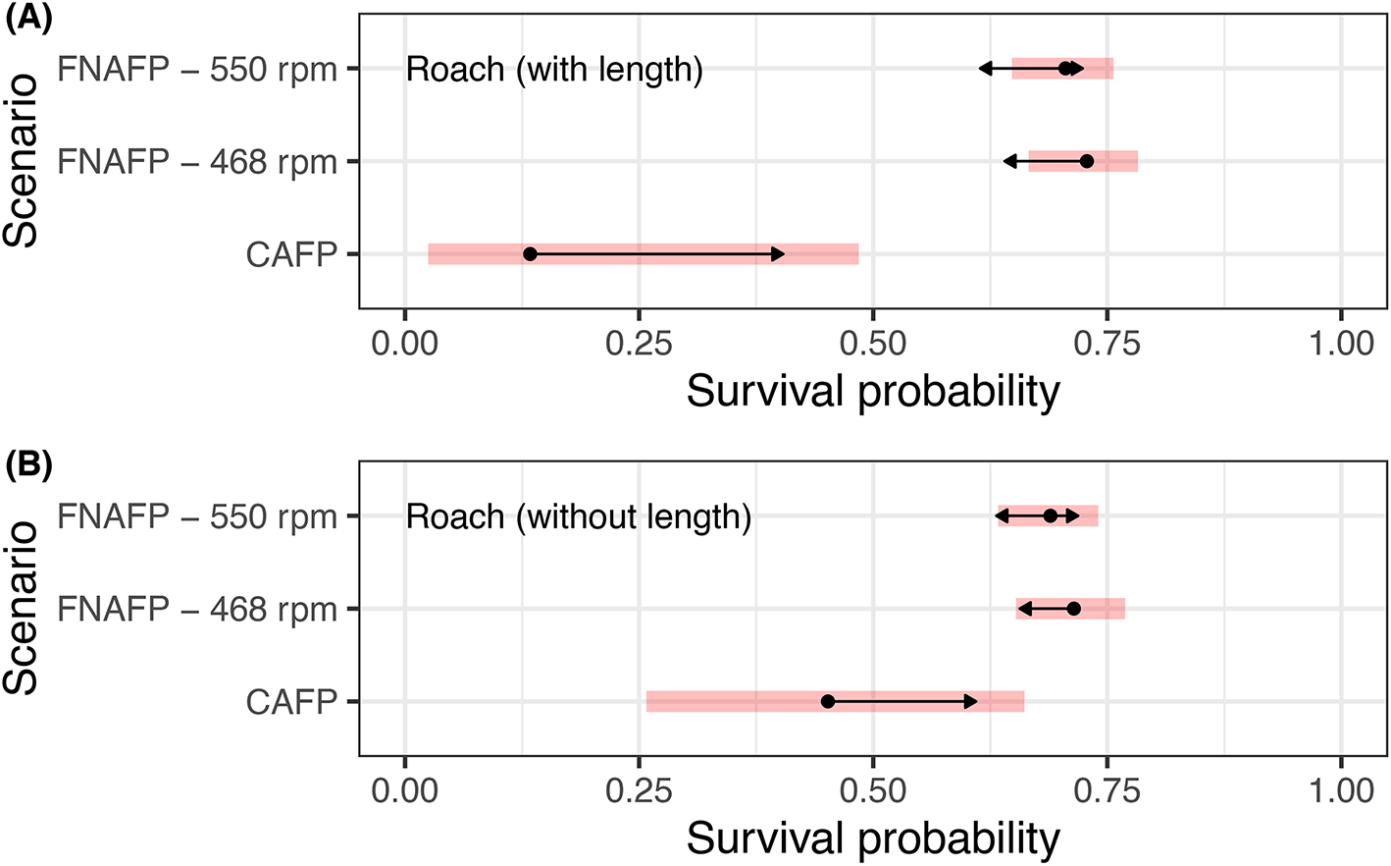
Model output of the most parsimonious logistic survival model for roach for which both the forced experiment and natural passage data was considered. In figure (**A**), fish length was accounted for in the model and estimates are for a fish with an average length of 164 mm. In figure (**B**), fish length was not considered. 95% confidence intervals are given per species and scenario as red bands. Non-overlapping arrows indicate a significant difference (*p* < 0.05). *FNAFP* Fairbanks Nijhuis Axial Flow Pump, *CAFP* Conventional Axial Flow Pump.

The capture of wild fish passing the CAFPs consisted of 2 perch, 16 bitterling, 33 roach, 2 stone moroko, 1 eel and 1 pike. To compare the survival rate between the CAFP and FNAFP, a logistic survival model was developed for roach with as response the survival rate and as explanatory variables the scenario (FNAFP at 468 rpm, FNAFP at 550 rpm and CAFP at 585 rpm), fish length and their interaction. The most parsimonious model did not retain the interaction of both factors (Supplementary Table S2). It is important to note that the length of the fish that passed the CAFP was significantly lower (*p* < 0.0001) than for the FNAFP (Supplementary Fig. S2). Hence, caution is advised when interpreting the results. However, whether length was included or not, the survival probability was always significantly lower (CAFP versus FNAFP at 468 rpm with length included: *p* $=$ 0.00090; CAFP versus FNAFP at 550 rpm with length included: *p* $=$ 0.0015; CAFP versus FNAFP at 468 rpm without length included: *p* $=$ 0.0098; CAFP versus FNAFP at 550 rpm without length included: *p* $=$ 0.0228) for the CAFP than for the FNAFP (Fig. 3 and Supplementary Fig. S4). The effect size of this difference was found to be larger when the length was considered in the model. When length was considered, the effect size difference between the FNAFP and CAFP was 2.74 (*p* = 0.0005) to 2.85 (*p* = 0.0003) while it was only 0.99 (p=0.0084) to 1.11 (*p* = 0.0035) when length was not considered. For all three scenarios, the survival probability decreased with increasing fish length.

#### Fish injury

The decision trees for the injuries were very similar to those of the survival probability (Supplementary Fig. S5 and Supplementary Fig. S6). This was to be expected as the injury class determined whether a fish was classified as dead or alive. Eel, control samples and roach (i.e. fish smaller than 198 mm) were most likely to have no injury (corresponding with injury 1), although the risk of an injury increased from eel, to control of bream or roach, to roach passing the pump. Bream on the other hand would on average most likely be severely injured unless it was a control sample (no injury) or an individual smaller than 340 mm subjected to a low rpm regime (slight injury) (Supplementary Fig. S7). The most likely class of injury for bream passing the pump was 3.2 (Incisions, cuts, decapitation). If, however, the length of the fish was lower than 340 mm, the most likely class of injury would be 2.3 (Light scratches, contusions, scale loss < 20% of body). Bream larger than 340 mm were most likely to have a 3.2 class injury, unless they were smaller than 400 mm and subjected to the lowest rpm regime in which case they were most likely to be diagnosed with a 2.3 class injury. For bream the most likely injury classes in decreasing order were 3.2, 2.3, 3.6 (Heavy contusion or bleeding), 3.1 (Scale loss), and 3.5 (Heavily injured gills or gill covers). Among the slight injuries 2.3 represented 96.6% of the cases, while the severe injuries were more evenly distributed among the different classes (3.2: 51.4% ; 3.6: 21.1%; 3.1: 14.6% and 3.5: 12.4%). At high rpm the most likely injury class was 3.2, while at low rpm the most likely injury class was 2.3 (Supplementary Fig. S13 and Supplementary Fig. S14).

For roach, the risk of an injury increased with length and rpm (Supplementary Fig. S8). For roach of average length (168 mm) the risk of an injury class 3.2 was significantly higher at 550 rpm than at 468 rpm (*p* $=$ 0.0050), while the contrary was true for injury class 2.3 (*p* $=$ 0.025) (Supplementary Fig. S12). However, unexpectedly, in overall roach larger than 170 mm and subjected to a low rpm regime were most likely to obtain a 2.3 injury while all other roach (also those smaller than 170 mm and subjected to a high rpm) were most likely to have no injury at all (Supplementary Fig. S8). For roach the most likely injury classes in decreasing order were 1, 2.3, 3.1, 3.5, 3.6 and 3.2. Among the slight injuries 2.3 represented 94.0% of the cases, while the severe injuries were more evenly distributed among the different classes (3.1: 37.9% ; 3.5: 25.4%; 3.6: 22.6% and 3.2: 14.1%).

**Fig. 4:**
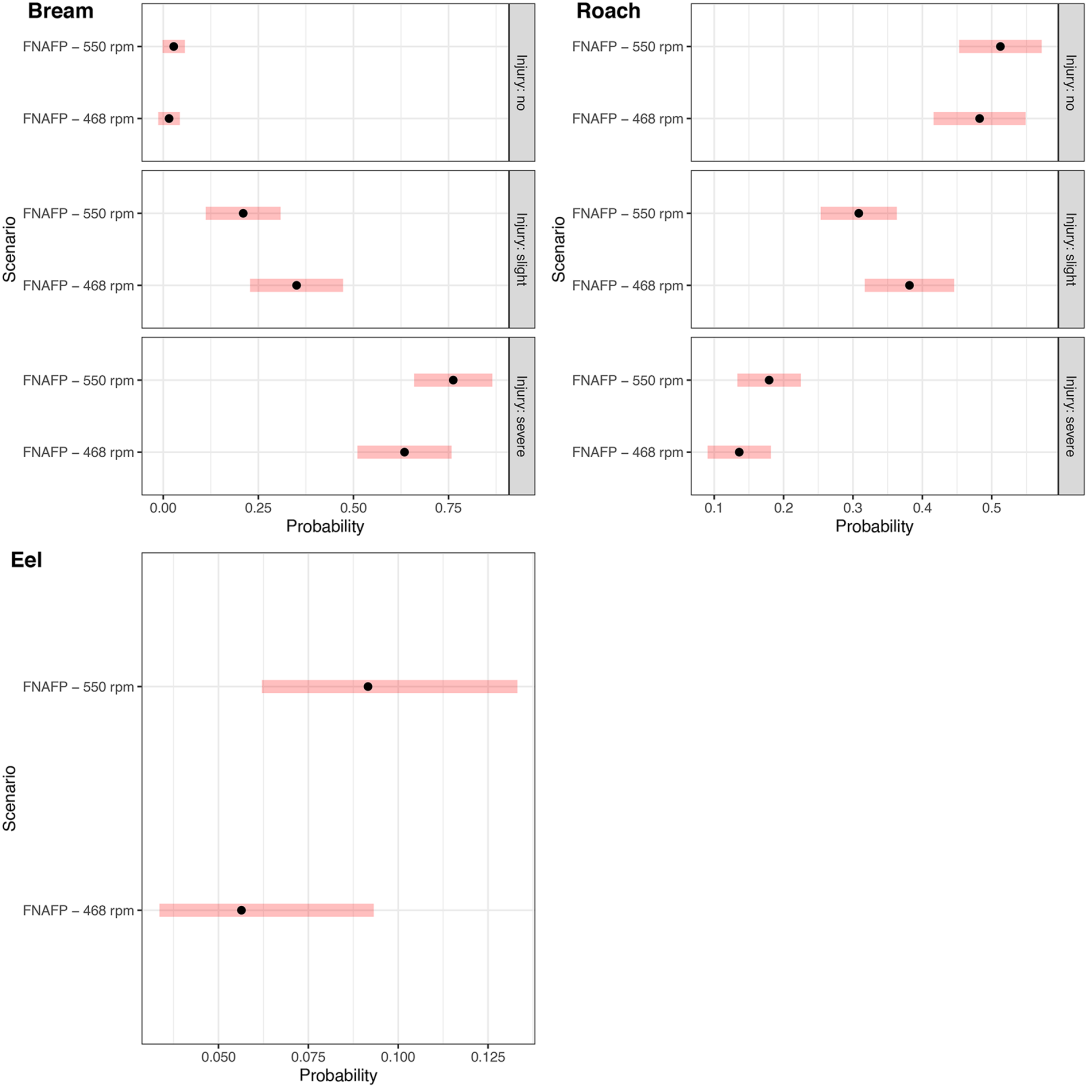
Model output of the most parsimonious multinomial injury models for bream, roach and eel depicting the probability a certain injury occurs. 95% confidence intervals are given per species and scenario as red bands. For eel, since there were only two observed injury classes (no versus slight injury), only the probability of a slight injury is depicted. *FNAFP* Fairbanks Nijhuis Axial Flow Pump.

The multinomial models provided additional insights. Although on average at the FNAFP high rpm regimes had more severe injuries and less slight injuries for roach and bream (Fig. 4), none of the differences were significant (Supplementary Table S3). For eel, only some slight injuries were observed which were on average more frequent at higher rpm regimes, though also not significant (*p* $=$ 0.27) (Fig. 4). Despite the absence of an effect of the rpm regime, there was a significant effect of fish length for both roach and bream (Supplementary Table S3). For both species, there was a relatively strong increase in the probability of obtaining a severe injury with increasing fish length (Supplementary Fig. S9 and Supplementary Fig. S10). The probability of no injury and the probability of a slight injury decreased slightly with increasing fish length. For bream the probability of no injury in function of fish length showed a convex trend, decreasing rapidly from 0.25 at 260 mm to almost 0 at 325 mm. The probability of a slight injury in function of fish length showed a concave, more gentile, decreasing trend. For roach the probability of no injury and the probability of a slight injury were not significantly affected by fish length (Supplementary Table S3). The probability of a severe injury in function of fish length showed a significantly increasing convex trend, indicating an increasingly high risk of a severe injury for larger fish (Supplementary Table S3).

**Fig. 5:**
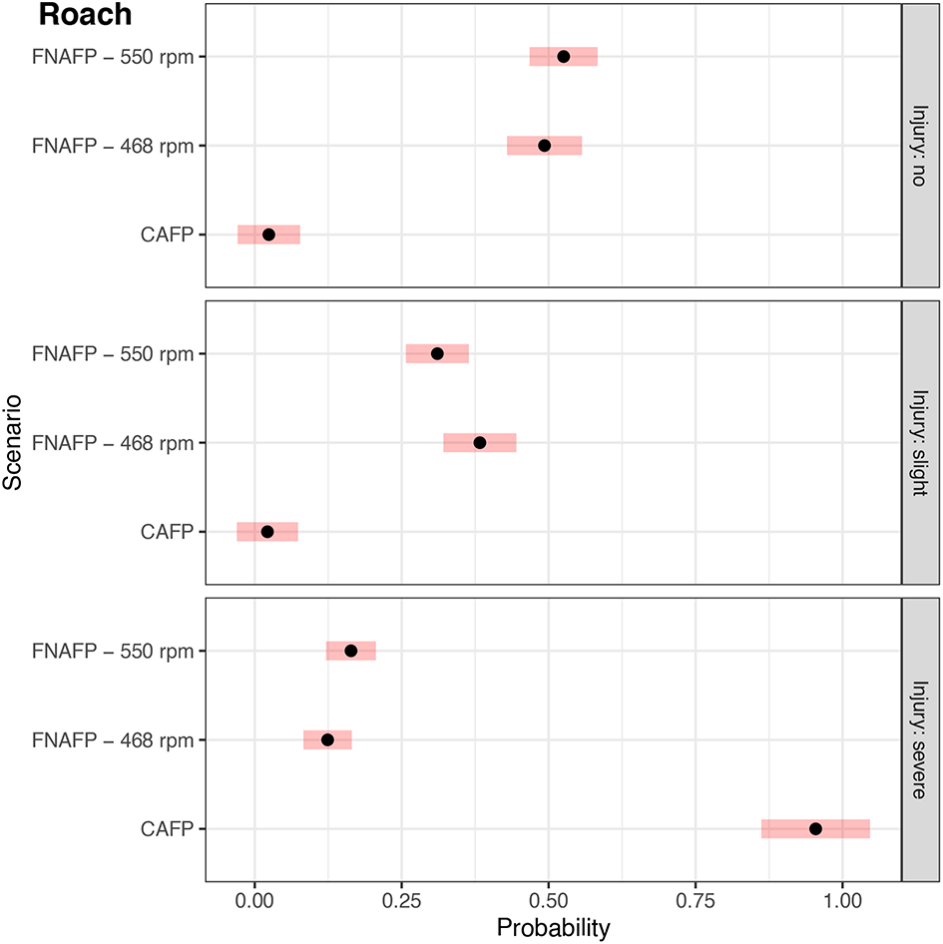
Model output of the most parsimonious multinomial injury models for roach depicting the probability a certain injury occurs for which both the forced experiment and natural passage data was considered. 95% confidence intervals are given per species and scenario as red bands. *FNAFP* Fairbanks Nijhuis Axial Flow Pump, *CAFP* Conventional Axial Flow Pump.

For roach that passed the CAFP instead of the FNAFP there was a significantly higher chance of a severe injury (*p* < 0.0001) and a significantly lower chance of a slight injury (*p* < 0.0001) or no injury (*p* < 0.0001) (Fig. 5 and Supplementary Table S2). Although there were only two roach larger than 150 mm that passed the CAFP, the model suggests that roach larger than 150 mm passing the CAFP had a very small chance of survival (6.8%), while the contrary was true for roach of the same length passing the FNAFP (91.5% for 468 rpm and 88.6% for 550 rpm) (Supplementary Fig. S11). Due to the small sample size of roach passing the CAFP and the multiple types of injury that need to be compared, statistical power was limited (Supplementary Fig. S17). Although on average class 3.1, 3.2, 3.3 and 3.4 were much more likely to occur in the CAFP than the FNAFP, only the higher chance of class 2.3 and class 1 in the FNAFP compared to CAFP were found to be significant (*p* < 0.0001) (Supplementary Fig. S12).

### Passive sensor assessment of barotrauma risk and shear stress

**Fig. 6:**
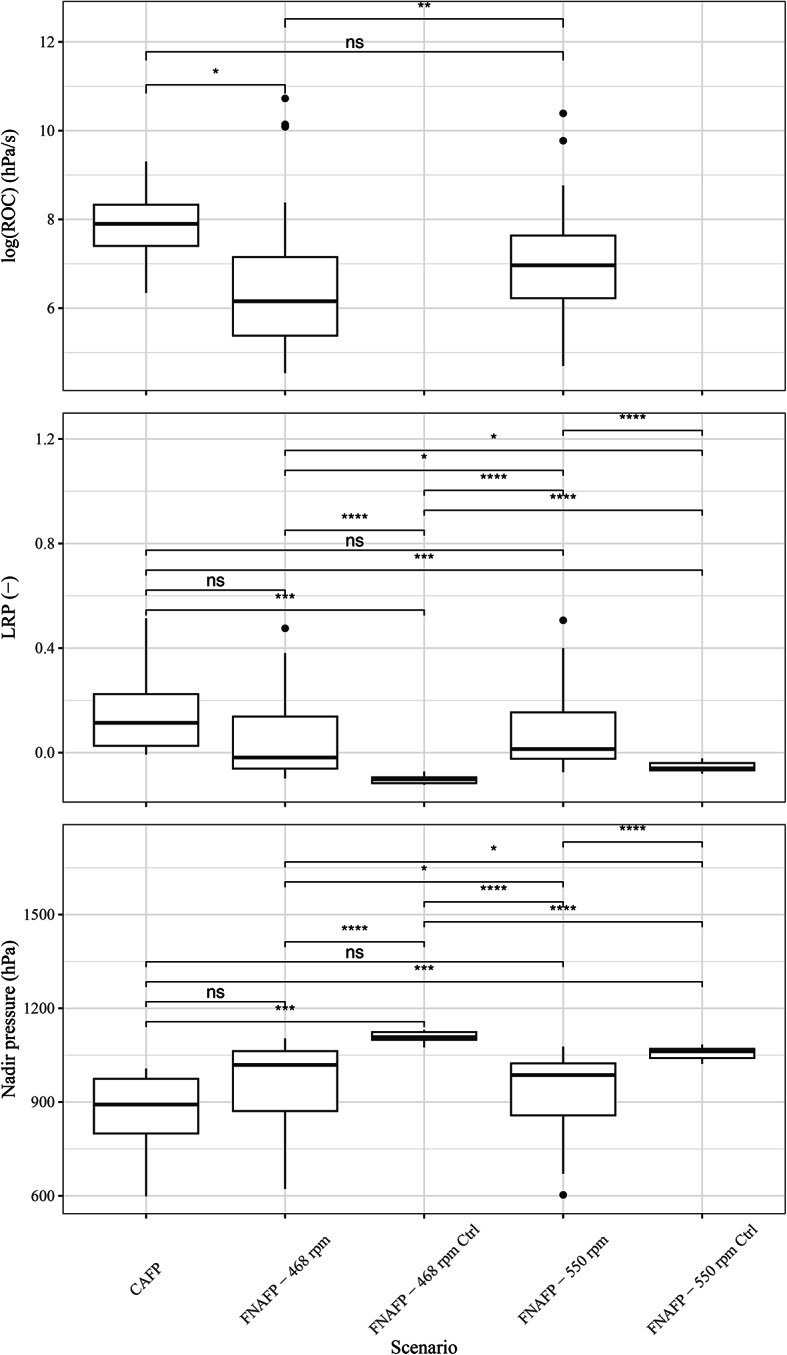
Boxplots representing the logarithm of ROC, LRP and nadir pressure per pump type and operating scenario. Pairwise Wilcoxon ranks sum tests with Bonferroni correction were applied. p-values were classified as non-significant or ns (> 0.05), *(0.05–0.01), **(0.01–0.001), ***(0.001–0.0001), ****(0.0001–0). *FNAFP* Fairbanks Nijhuis Axial Flow Pump, *CAFP* Conventional Axial Flow Pump, *Ctrl* Control, *LRP* Log Ratio Pressure change, *ROC* Rate Of Change.

**Fig. 7:**
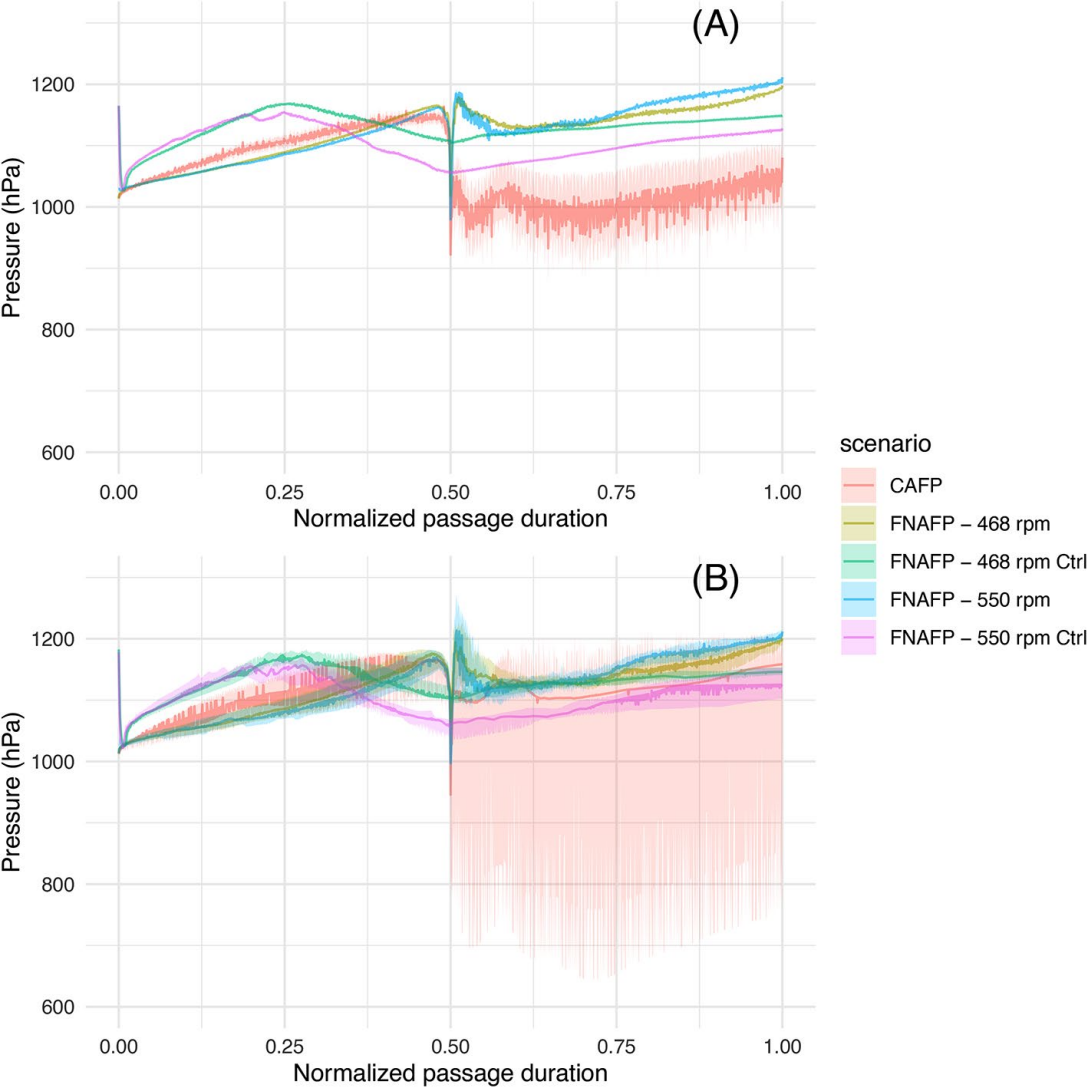
(**A**) Time series plots of pressure, averaged over all sensors with 95% CI. (**B**) Time series plots of pressure, median over all sensors with Q25% and Q75%. *FNAFP* Fairbanks Nijhuis Axial Flow Pump, *CAFP* Conventional Axial Flow Pump, *Ctrl* Control.

**Fig. 8:**
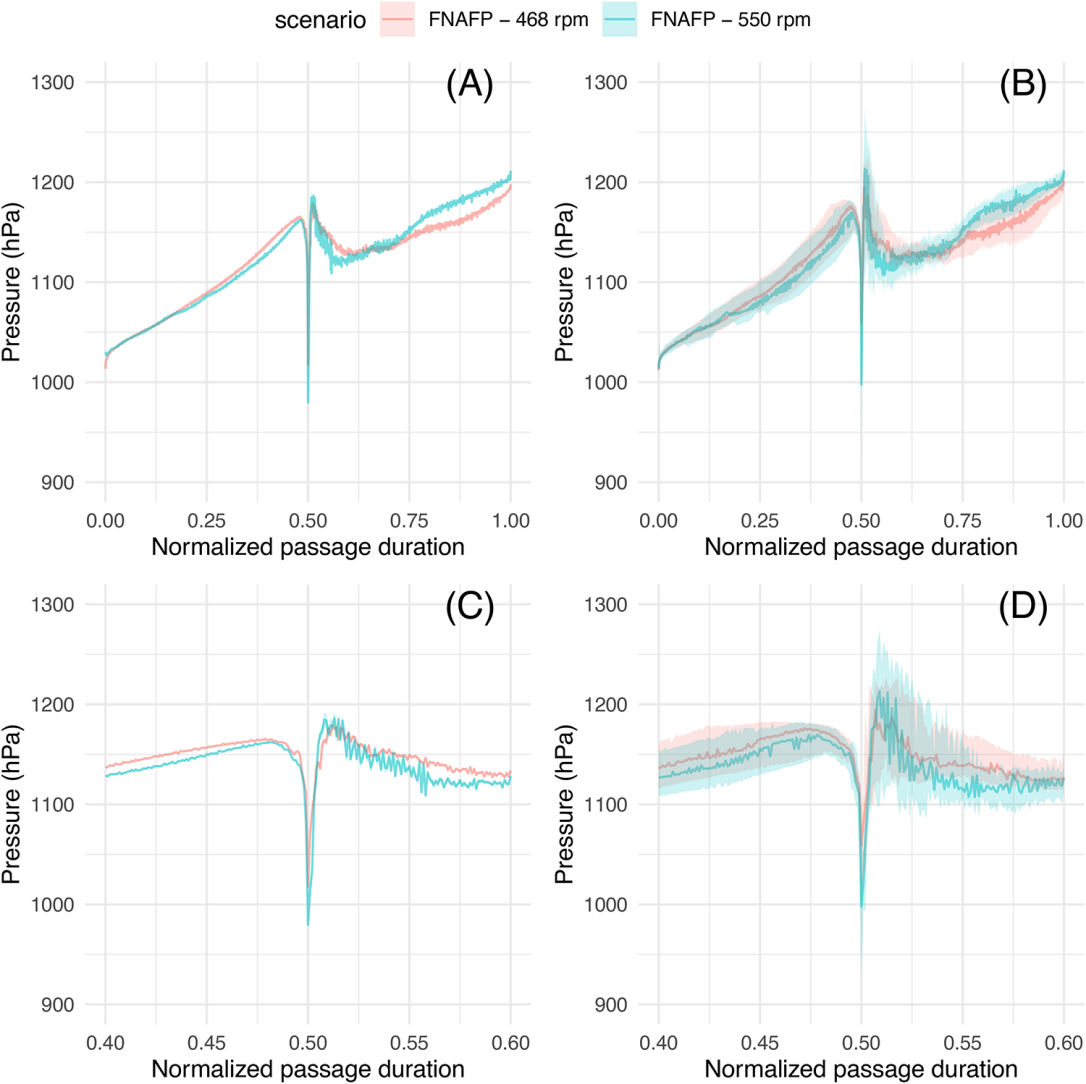
(**A**) Time series plots of pressure, averaged over all sensors with 95% CI. (**B**) Time series plots of pressure, median over all sensors with Q25% and Q75%. (**C**) Time series plots of pressure, averaged over all sensors with 95% CI. Focus on the nadir (relative time between 0.4 and 0.6 with 0.5 the moment of the nadir). (**D**) Time series plots of pressure, median over all sensors with Q25% and Q75%. Focus on the nadir (relative time between 0.4 and 0.6 with 0.5 the moment of the nadir. *FNAFP* Fairbanks Nijhuis Axial Flow Pump, *CAFP* Conventional Axial Flow Pump.

The nadir pressure, rate of pressure change (ROC) and log ratio pressure change (LRP) were used to estimate the risk of barotrauma-related injury and survival rate based on the passive sensor data collected in both pumping stations (Table 1). Scenarios with higher LRP values (here the acclimation pressure was taken as 1000 hPa) are known to present fish with an increased risk of barotrauma^27^. As expected, the control samples had a significantly lower LRP than the samples passing through the pump (Fig. 6 and Supplementary Fig. S15). The pump at 550 rpm had significantly higher LRP (*p* $=$ 0.032) and ROC (*p* $=$ 0.0027) values than the pump at 468 rpm (Figs. 6, 7 and 8). Although the CAFP had on average a clearly higher LRP than the FNAFP regardless of the rpm, no significant differences (*p* $=$ 0.064 and *p* $=$ 0.20 for the comparison with the FNAFP at low and high rpm respectively) were found (Fig. 6). This may be the result of the relatively low number of sensors used in the axial pump.

**Table 1:** Summary of the three pressure-based parameters (reference atmospheric pressure 1000 hPa) from the BDS passive sensor deployments at the Devil’s Hole pumping station in Belgium. The parameter values are reported as their ensemble means ± their standard deviations (SD). *FNAFP* Fairbanks Nijhuis Axial Flow Pump, *CAFP* Conventional Axial Flow Pump, *LRP* Log Ratio Pressure change, *ROC* Rate Of Change.

Pressure-Based Parameter	CAFP, 585 rpm (n = 6)	FNAFP, 468 rpm (n = 64)	FNAFP, 550 rpm (n = 51)
Nadir (hPa)	854 ± 164	964 ± 137	933 ± 126
LRP (–)	0.174 ± 0.210	0.048 ± 0.155	0.079 ± 0.147
ROC (hPa/s)	4008 ± 4118	2657 ± 7604	2501 ± 5248

When the average and median are taken of the different time-normalized pressure time series, it becomes visually clear how the nadir pressure is lowest for the CAFP, followed by the FNAFP at high rpm and the FNAFP at low rpm (Figs. 7 and 8). For the FNAFP, it was found that the pressure steadily increases immediately after sensor injection. Important to note is however that this linear registration smooths away any signals that do not occur at specific moments relative to the reference points (i.e. at the moment of injection, the nadir and the exit to tailwater).

**Fig. 9:**
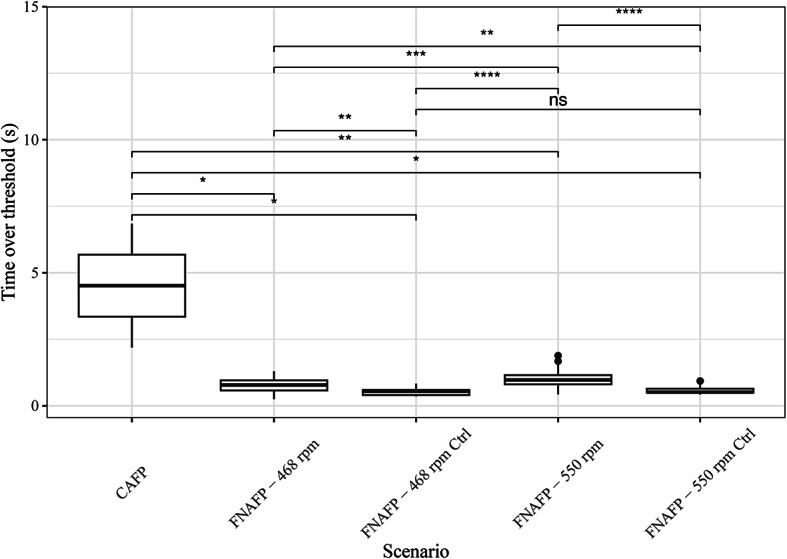
Boxplots representing the duration the shear strain threshold is exceeded per pump type and operating scenario. Pairwise wilcoxon ranks sum tests with Bonferroni correction were applied. p-values were classified as non-significant or ns (> 0.05), *(0.05–0.01), **(0.01–0.001), ***(0.001–0.0001), ****(0.0001–0). *FNAFP* Fairbanks Nijhuis Axial Flow Pump, *CAFP* Conventional Axial Flow Pump, *Ctrl* Control.

The duration of harmful shear strain exposion (i.e. the shear stress that fishes endure) was significantly larger in the CAFP than in the FNAFP (*p* $=$ 0.018 and *p* $=$ 0.0017 for the comparison with the FNAFP at low and high rpm respectively) (Fig. 9). For the FNAFP, shear strain exposion duration was significantly higher at 550 rpm than at 468 rpm (*p* $=$ 0.00011). And for both FNAFP scenarios the shear strain exposion duration was larger than for the control samples. The control samples themselves had the same shear strain exposion duration. The average and maximal mean strain rate showed a mainly opposite pattern to that of the duration of harmful shear strain exposion, with the CAFP having the lowest average and maximal strain rate (Supplementary Fig. S16). Differences between the FNAFP scenarios and their corresponding control samples were non-significant.

## Discussion

### How fish-friendly is the FNAFP?

Although in this study the FNAFP outperformed the CAFP in terms of fish safety, the survival rate of the FNAFP for roach and bream were substantially lower than the 97% postulated by the pump manufacturer^23^. The manufacturer’s claim regarding the 100% survival rate of eel on the other hand, was in line with the results of this study^23^. As indicated earlier, hitherto there have only been two studies on the survival rate of the FNAFP and the results of these studies should be treated with care due to the small sample size, low recapture rate and questionable assumptions regarding the origin of the injuries^23,24^. Bierschenk et al., noted 0% mortality for roach passing through the FNAFP, but also indicated that these results “should be interpreted cautiously because of the low recapture rate” as only 2 out of 200 fish (1%) were recaptured^24^. Recapture rates of the common nase and perch were only 0.4% (1 fish) and 8% (11 fish) respectively and only 13 wild fish were captured at the outlet of the pump. The study of Vriese et al. also suffered from small sample sizes (i.e. 32 roach, 28 bream and 25 eel) and an unexpected injury source (i.e. fish were blown with great force into the nets after the pump)^23^. All 16 injuries to the roach and 3 of 5 injuries found on bream were attributed to forced contact with the net. As no standardized injury protocol was used and documentation regarding the difference in types of injuries caused by the net versus the pump was very limited, these results remain challenging to interpret. In the current study, control samples were used to assess any effect the handling before and after the pump might have had. Since there was only one fish with an injury out of 326 control samples the risk of wrongly assigning an injury or death to handling instead of pump passage can be considered negligible. Finally, the characteristics of the pump and the conditions at which the pumps were operated differed, with the 600 mm diameter FNAFP used by Bierschenk et al. operating at 495 rpm and having a discharge of 9 m$^{3}$s$^{-1}$ and the 800 mm diameter FNAFP used by Vriese et al. operating at 333 rpm and having a discharge of 1.35 m$^{3}$ s$^{-1}$^23,24^. In this study a 600 mm FNAFP operating at 468 and 550 rpm and having a discharge of 0.76 and 1.10 m$^{3}$s$^{-1}$ respectively, was used. The dimensions, which are fixed and therefore difficult to consider in an experimental setting, and operational conditions of any pump are crucial to acknowledge when evaluating its fish-friendliness. Results for one pump cannot be extrapolated to any other pump without considering its physical parameters and operational characteristics, even if both pumps operate under the same general working principles. Given the limitations of the earlier mentioned studies, we believe that the current study should serve as a suitable baseline for validating the fish-friendliness of the FNAFP and highlight the need for additional studies under different conditions. Though we were able to establish that the FNAFP had a survival rate of 100, 70 and 24% for eel, roach and bream respectively, the lack of regulations and rules on the requirements a fish-friendly pump should meet prevents us from postulating whether the FNAFP is to be considered fish-friendly or not. Unfortunately, the question whether the FNAFP, and any other pump for that matter, is fish-friendly therefore remains mainly of a rhetorical nature. International legislation supported by sound scientific results that prevents companies from using the “fish-friendliness” label in vain is needed to guide decision makers responsible for the purchase, installation and maintenance of new pump systems.

### Which parameters affect survival rate and injury?

Pump passage, species, fish length and rpm were found to affect survival rate and injury. As expected, the most important parameter to affect the survival rate and the risk of severe injury for roach and bream was whether a fish passed the pump or not. As mentioned, only one fish of the control samples was severely injured out of 326 fish. It is therefore reasonable to consider the effect of handling to be minimal and the damaging effect of the pump for roach and bream as significant. Since 100% eel survival was observed for both the control samples and the pump passing samples, the FNAFP operating under the investigated conditions can be considered safe for eel passage. The second most important factor to affect the survival rate and severe injury risk for roach and bream during pump passage was the fish total body length and/or species. As bream were always larger than roach and larger fish seemed to be more susceptible to injury, it cannot be said with certainty whether fish length alone or fish length in combination with other species-specific properties such as body shape, scale type, swim bladder morphology and behavior were responsible^25,28^. Fish length may be a factor which negatively affected the survival of roach in the CAFP. Baumgarter described how fish smaller than 50 mm and larger than 200 mm passing axial pumps were most susceptible due to shear stress and strike, respectively. However, Baumgarter did not assess fish length in combination with species which may bias the results, as it can be the case that either fish length, species-specific behaviors or a combination of the factors affect survival rates after pump passage^29^.

Finally, though the effect of increased rpm was not significant, it was characterized by a lower average survival probability which was most pronounced for bream. If roach was severely injured, the injury most likely consisted of scale loss, while for bream it was most likely related to incisions, cuts or decapitation. For both bream and roach the risk of incisions, cuts or decapitation increased with increasing rpm, but the effect was most pronounced for bream. Regardless of rpm, for roach the most important class of severe injury remained scale loss. However, for roach passing the CAFP the most common class of severe injury were incisions, cuts or decapitation.

Unfortunately, since experiments per fish were done within a day (eel: 23/10/2018, roach: 20/11/2018; bream: 18/12/2018) each time, it was not possible to assess the effect other environmental variables such as water temperature might have had on the risk of injury and survival rate. Though environmental conditions are known to affect injury risk^5,30^, to be able to assess the effect of environmental variables the experiment would have needed additional replicates spread over time to cover the variability in environmental conditions. In a close by river (Ede) known to have similar water temperatures as the studied system, average daily water temperature was 10.4$\,^{\circ }\hbox {C}$, 4.9$\,^{\circ }\hbox {C}$ and 6.5$\,^{\circ }\hbox {C}$ during the eel, roach, and bream experiment respectively. Since higher temperatures are known to increase fish metabolism and activity they might increase the risk of injury via collisions^30^. Furthermore, when fish are outside the optimal temperature range (roach: 12–25 $^{\circ }\hbox {C}$; bream: 10–26 $^{\circ }\hbox {C}$; eel: 4–20 $^{\circ }\hbox {C}$) fish might become more stressed and in turn more vulnerable to mechanical injuries^5,31^. During the study, water temperature was only optimal for eel as it was considerably higher than during the bream and roach experiments, making the latter two fish species potentially more susceptible to injuries. However, the higher temperature during the eel experiment might also have caused them to be more active than bream and roach were, potentially counteracting the first effect. Estimates regarding the effect of environmental conditions remain limited especially when they need to be assessed together with operating conditions of the pumps themselves. Ideally, sufficient replicates to cover the different sources of variability would be advised though the cost of such extensive experiments would be difficult to justify to an ethical committee.

### What are the sources of injury and mortality?

Identifying the source of the injuries and mortality of fish passing through pumping stations remains challenging due to methodological constraints of observing the fish trajectory and the inherently complex and dynamic flows within the structures^20^. The state of fish entering and leaving the structure can be assessed, though where exactly the fish obtains its injury cannot be determined^32^. Furthermore fish are more likely to be injured by a combination of elements and multiple injury types may mask each other^5^. Passive sensors and backpack sensors might give us some idea of the conditions that fish endure, but they can never be a full substitute to what the fish experiences due to the physical differences between passive sensor bodies and those of the live fish. In hydraulic structures, the known sources of injury are shear stress (due to the force exerted by opposite movement of water), pressure changes (rapid decompression), turbulence, aeration and collisions with rigid objects and impeller strike^32^. In pumps injuries due to strike, collisions and pressure changes are assumed to be the most common^3,33^.

Barotrauma is the result of fish being exposed to rapid decompression events, which are dependent on the design, operation, head, submergence and flow rate of the pump and the trajectory of the fish^27^. In this study, The LRP (Log Ratio Pressure change) for the CAFP, the FNAFP at low rpm and the FNAFP at high rpm were on average 0.17, 0.048 and 0.079 respectively, and therefore it is unlikely that barotrauma would be the main source of injury in both the FNAFP and CAFP^34^. A controlled pressure study with the physostome Chinook salmon suggested that the aforementioned LRP values would correspond with a survival probability of more than 99%^34^. Eel, bream and roach are also considered physostome fish, meaning that they have a duct allowing them to transfer air from the swim bladder to the gut and as such respond to pressure changes, making them less vulnerable to barotrauma^35^. Though a controlled study on silver perch juveniles revealed a few cases of kidney haemorrhage at 0.17 LRP, it should be noted that this species is ill-adapted to pressure changes altogether^36^.

Although the observed damage to the gills, operculum, and eyes may have been caused by barotrauma, it is much more likely that these were caused by contact events with the interior surfaces of the pump and impeller strikes. Injuries which are typically characteristic for barotrauma (i.e. stomach eversion and swim bladder rupture) were not observed, while injuries common to collision and strikes (i.e. bruising, scale loss, fin damage, amputation and decapitation) were present^20^. In addition, although fish size will, to some extent, determine the trajectory of the fish, the size itself is not a dominant factor affecting the risk of injury by barotrauma. Since the risk of injury and mortality in this study was found to be highly size dependent it is unlikely that barotrauma was the main cause behind the observed injuries. Indeed, the risk of collisions, blade strikes and shear stress is typically more size dependent^32^. How shear strain is experienced by fish is determined by both the magnitude and duration of the harmful shear strain exposion^33^. While the duration of harmful shear strain exposion decreased from the CAFP to the FNAFP at high rpm to the FNAFP at low rpm, the observed average and maximal mean shear strain showed a mainly opposite pattern. This makes it difficult to determine whether shear stress was an issue in either pumps. However, since shear stress is known to be more harmful for smaller fish^37^ and given that the risk of injury and mortality increased significantly with fish length for both bream and roach, it is unlikely that shear stress was the main cause of observed injury and mortality. Furthermore, since shear stress is known to cause a wide range of types of injury^32^, we would have expected a much wider range of prevalent injury types under a shear-stress-dominated environment. Therefore, it is much more likely that collisions and blade strikes were the dominant sources of harm^27,32^.

For both the FNAFP and CAFP, strike and collision appear the most likely sources of injury and mortality which corroborate the results of previous studies at other pump installations^3,38,39^. Given that for both pumps the trajectory and operating conditions are similar, it is most likely the smoothed edges of the impeller and large center opening of the FNAFP that results in fewer incidents of strike and collision, respectively^38^. The reduced risk of strike seemed to have been the strongest contribution of the FNAFP over the CAFP. Indeed, the most frequent severe injuries that roach obtained in the CAFP were incisions and amputations which were most likely the result of strike by the impeller, while for roach in the FNAFP these were the least frequent severe injuries. Though for roach collisions seemed to be less frequent and of a less severe nature in the FNAFP compared to the CAFP, the many instances of minor scale loss and bruising in the FNAFP suggest collisions are still an issue and most likely the most important source of injuries given that major scale loss was the most frequent type of severe injury roach obtained in the FNAFP. It seemed that in the FNAFP however, strike seemed to become more prominent if rpm increased. This was especially the case for bream, where at low rpm minor scale loss and bruising were the most frequent type of injury while at high rpm severe incisions and amputations were most common.

## Conclusion

Though the FNAFP outperforms the traditional CAFP in terms of fish-friendliness due to its smoother impeller edges and larger center opening which reduces the risk on strike events, its ability to let fish pass safely remains strongly dependent on the species, fish characteristics and operating conditions. The complexity of interacting factors in combination with the difficulty to pinpoint observed injuries to likely sources warrants a standardized assessment, or at least a checklist of required elements, when evaluating and comparing pumping installations. Besides agreed-upon protocols, consensus on what criteria a fish-safe or fish-friendly pump should meet to be classified as such are desperately needed to provide water managers with some footing to make informed choices regarding pump purchases, installations and operation. More concretely, we argue that a standardized database bringing together studies on the fish safety of different pump installations operating under different conditions would allow for the distillation of evidence-based rules, foreseen with uncertainty clauses, to guide water managers in their decisions. Finally, though the conditions of baseline studies with a sound design, like this one, are unlikely to be a perfect match to any real life situation water managers are faced with, they can still inform on what might be extrapolated but also caution against what cannot and underline the need for additional testing when in doubt.

## Methods

### Study area and description of the pump installations

**Fig. 10:**
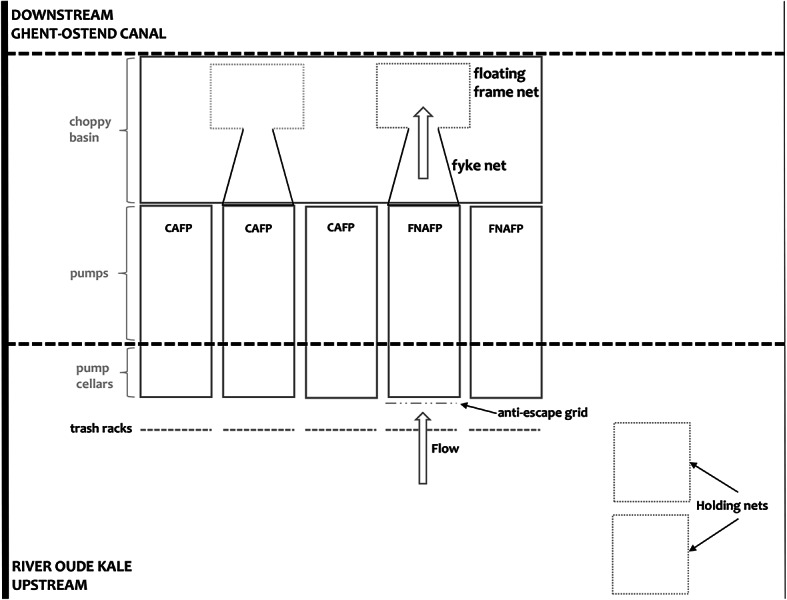
Schematic drawing (top view) of the Devil’s hole pumping station and the experimental set-up with an indication of flow direction.

The Devil’s Hole pumping station on the lowland River Oude Kale in Vinderhoute (Belgium) (Latitude 51.087184$^{\circ }$ (N), Longitude 3.656618$^{\circ }$ (E), WGS84) pumps excess water from the Oude Kale to the Ghent-Ostend-Canal (Fig. 10). It is fitted with three classic axial flow pumps (CAFPs) and two Fairbanks Nijhuis axial flow pumps (FNAFPs).

The CAFPs of the pumping station have a four-blade propeller, a discharge nozzle diameter of 600 mm, a suction nozzle diameter of 750 mm, an impeller diameter of 580 mm, a blade angle of 19$^{\circ }$ and a fixed speed of 585 rotations per minute (rpm). Each CAFP has a discharge capacity of 1 m$^{3}$s$^{-1}$.

The FNAFP was developed by FishFlow Innovations in collaboration with Nijhuis Pumps^23^. The principle of this fish-friendly pump is based on an adjustment of the impeller shape and the guide vanes of the pump. They are characterized by (1) a design derived from the tube screw of FishFlow Innovations, (2) a larger ball diameter compared to conventional propellers, and (3) rounded impeller and guide vane edges to prevent cuts^40^. These adjustments should ensure that fish can pass the pumps without injuries. In addition, it should make the propeller cavitation-free and therefore less noisy, which ensures that fish do not experience the pump as a barrier to migrate. The FNAFP has the same nozzle diameter as the CAFP (600 mm) but can vary its speed from 293 rpm (25 Hz; 50%) to 585 rpm (50 Hz; 100%), using a frequency converter. Thus, the discharge capacity varies between 0.47 m$^{3}$s$^{-1}$ and 1.1 m$^{3}$s$^{-1}$. The blade angle is fixed at 30$^{\circ }$. In this study, fish and barotrauma detection sensors (BDS) were assessed at two different operational scenarios (i.e. rotation speeds): (1) at a normal working point of 550 rpm (47 Hz) and (2) at a reduced working point of 468 rpm (40 Hz) (Table 2). For the CAFPs, no live fish were actively added due to ethical reasons as the CAFPs are expected to have a very low survival rate^41,42^. Instead, though less statistically powerful in comparing differences between pumps and across operational settings, natural passage via the CAFPs was recorded.

**Table 2:** Number of fish intended (Start fish), number of fish captured (End fish), recapture percentage (Rec. %) for each scenario of the FNAFP (Pump, rpm and Type) per species and average (Av.) length and weight. Note that for bream one scenario had a recapture rate of more than 100%. This is because fish intended for a previous scenario ended up in the subsequent scenario, resulting in a larger amount of fish than was initially provided for the subsequent scenario. In total 300 bream, 617 eel and 909 roach were used.

Species	rpm	Type	Start fish	End fish	Rec. %	Av. length (mm)	Av. weight (g)
Bream	468	Control	50	50	100	406	1250
Bream	468	Pump	155	97	63	380	963
Bream	550	Control	45	44	98	407	1257
Bream	550	Pump	52	109	210	369	823
Eel	468	Control	50	47	94	639	453
Eel	468	Pump	300	256	85	633	436
Eel	550	Control	50	47	94	638	436
Eel	550	Pump	300	267	89	631	432
Roach	468	Control	100	97	97	172	85
Roach	468	Pump	400	343	86	167	78
Roach	550	Control	50	41	82	165	74
Roach	550	Pump	500	428	86	168	78

### Fish deployment and analysis

#### Fish data collection

Based on the Dutch NEN 8775 standard, (which describes a method for the determination of fish safety of pumps, Archimedes screws, and confined water turbines used in pumping stations and hydroelectric plants) three different hatchery-raised species were selected for evaluation of the FNAFP, namely European eel (*Anguilla Anguilla*), bream (*Abramis brama*) and roach (*Rutilus rutilus*)^43^. The eel was provided by hatchery Borremans from Berlare (Belgium) while the roach and bream were provided by hatchery Vandeput from Zonhoven (Belgium). Although the aim was to have at least 300 individuals per species per scenario (Supplementary Fig. S17), early testing showed a low survival rate for bream and therefore only approximately 100 individuals of bream were used per scenario (excluding the control scenarios for which we aimed to have approximately 50 fish) (Table 2). Fin cuts were used to distinguish between fish from different scenarios. To acclimatize to the water of the river where the pumping station is located and to recover from any stress caused by transport, the test fish were kept in floating frame nets upstream of the Devil’s Hole pumping station for at least 24 h (Fig. 10). Knotless nets were used to prevent injury from friction/abrasion. The nets were covered with soft netting material to prevent the fish from jumping or being predated by e.g. birds. The densities in the floating frame nets were comparable to those at fish farms. The use of the experimental animals is in accordance with Belgian legislation (EC approved by Ethical committee: case number ECINBO-010). Experiments were performed and reported in compliance with the ARRIVE guidelines^44^.

Experimental trials with European eel, roach and bream were conducted in October 2018, November 2018 and December 2018, respectively. The length distribution of the fish used during the experiments should reflect the length distribution fish would have during the life stage they are most likely to pass pumps (i.e. spawning migration)^43^. However, in practice, researchers are strongly dependent on a few nearby suppliers who only have a limited availability of different length classes. The average fish length of the three different species used in this study was significantly different (see results), making it impossible to discern whether fish length or other species attributes caused differences in survival rate. However, since in nature the population average of the three species is also significantly different, pursuing a comparable average length among species would have led to results that are hard to extrapolate to real life situations due to a mismatch between the population average and the average of the experiments. Therefore, the statistical comparison between species should always consider the likely presence of confounding factors and results should be extrapolated with care as attributes like fish length can have an important effect.

Prior to entering the pumps, the health of each fish was assessed and potentially damaged fish were excluded from the tests. Next, the fish were transferred into a well-aerated transport basin and released with dip nets at the calm water surface of the pump cellar. A grid about five meters upstream of the pump prevented the animals from escaping to the Oude Kale. The test animals were thus introduced in the calm upper water layer at a considerable distance from the pump and could therefore reorient and approach the pump in a natural way. Fish were captured after passage via fyke nets. Control animals were added to the fyke nets behind the pump to evaluate possible injuries caused by handling or related to the experimental set-up.

After pump passage, the nets were emptied and all fish were immediately transferred into large aerated holding 90-L tanks. The fish were checked for abnormal swimming behaviour (floats, back swimmers,...). The condition (dead or alive) of each fish and their physical status based on visible external or internal injuries were examined, according to the classification of the NEN guidelines^43^. Fish injuries were divided into three categories: (1) injury free; (2) minor superficial scratches (2.1), red or damaged eyes/fins (2.2), or minor scale loss (2.3); (3) Scale loss (> 20% of body surface; 3.1), Incisions, cuts, decapitation (3.2), fractures (3.3), heavily injured or missing eyes (3.4), heavily injured gills or gill covers (3.5), heavy contusion or bleeding (3.6), Aberrant behavior (3.7). In the case of decapitated or incomplete individuals, the number of fish was determined only by counting the number of heads. Pictures were taken of fish with injuries of categories 2 and 3. Fish with barotrauma have one or more of the following characteristics: damaged or ruptured swim bladder, air bubbles in the eyes, and/or air bubbles in tissues. If the fish was intact, fish characteristics were measured: body mass (to the nearest g) and total length (to the nearest mm). Fish with injuries of category 3 were euthanized by administering a clove oil (Sigma-Aldrich) overdose (3 ml/l). After completing the test, healthy, undamaged, and slightly damaged fish were stored in the floating frame nets next to the Devil’s Hole pumping station to study delayed mortality. After 24 h, delayed mortality was examined and dead fish were removed from the nets. The day after, delayed mortality and abnormal swimming behaviour (as, for instance, the result of unobserved internal injury) were examined. All surviving fish were released in the Ghent-Ostend Canal after permission of the Agency for Nature and Forests (ANB) (Fig. 10). If internal injuries could be observed directly (for instance via signs of apparent internal bleeding) or indirectly (via the behaviour) within 24 h after the experiment they were noted and classified. Though we considered this to be unlikely, some internal injuries might have gone unnoticed and might have negatively affected fish survival leading to an underestimation of the mortality. To generalize the underestimation of mortality at different durations-since-the-experiment is difficult as it is known to vary substantially between and even within pump systems^24^. A threshold is therefore not available though most experiments keep fish for 24 h to determine delayed mortality.

The damage to cyprinids and eels after natural and unforced migration through propeller or classic axial flow pumps is typically very high^42^. Even though each pumping station is configured differently, we can assume that for this study a large number of the test animals would experience considerable discomfort during and after pump passage through a CAFP of the Devil’s Hole pumping station. It was therefore not ethically justifiable nor allowed by the ethical committee (based on earlier results at a similar pumping installation^42^) to determine the survival rate of fish by forced passage through the CAFPs. Instead, the unavoidable natural passage of fish was recorded when the CAFPs were operational. Therefore, a fyke net was attached to the outflow of one of the CAFPs (Fig. 10). In addition, BDS were passed through the CAFP and FNAFP, measuring the physical conditions during the passage. BDS and fish were recovered via the fyke nets.

#### Fish data processing

The injuries were grouped in injury classes that ranged from no injury (category 1), to slightly injured (category 2), to severely injured (category 3). The most severe injury class observed for an individual determined the injury class the individual was assigned to. Fish were classified as dead in case they were observed as dead, dying or alive with a severe injury. During the trial, the pump cellar of the FNAFP was observed from above to determine whether there were fish swimming against the current near the screen, thereby avoiding being pumped. Fish that lingered in the pump cellar of the FNAFP were often only pumped during the next trial which had a different pump rotational speed. Therefore, these fish were assigned to this subsequent scenario. Because of this there can be a difference in the theoretical scenario fish were assigned to and the actually observed scenario. Assigning fish to the scenario at which they were observed leaving the FNAFP was justifiable as pumped fish were immediately netted in the strong turbulent outflow of the pump. Hiding in the short and turbulent area downstream of the valve was practically impossible. Fish with outlying length values, i.e. values that fell outside of 3 times IQR (interquartile range) below Q1 or 3 times IQR above Q3, were removed. For roach, 6 extreme outliers were removed. For the other species no measurements were removed.

#### Fish data analysis

For all analyses the factors rpm (as discrete variable: 468 versus 550 rpm) and fish length (as continuous variable) were considered. In case the entire dataset was considered (i.e. all species), the factor species was also included in the analyses. Data preprocessing and analysis were done using the R software (version 3.6.2, R Developer Core Team, R Foundation for Statistical Computing, Vienna, Austria).

To find out to what extent the fish length distributions of the different scenarios of the FNAFP differed, ANOVA models were constructed with the response being the fish length and the variables being the different scenarios. If a significant difference (*p* < 0.05) was found between the scenarios, post-hoc pairwise comparisons with Tukey correction were performed. 95% confidence intervals were calculated and visualized. Analyses were performed per species.

To assess the survival rate for the different scenarios of the FNAFP, decision trees were developed for the entire dataset and for each species separately^45^. These provide an intuitive and clear overview of the data and underlying patterns. The state of each fish (alive = 1 versus dead = 0) was used as response. The explanatory variables were species (discrete), rpm (discrete) and fish length (continuous). Additionally, to provide a more detailed assessment of factors affecting the survival rate, logistic models with Bernoulli response were developed for each species separately. The state of each fish (alive = 1 versus dead = 0) was used as response. The explanatory variables of the full model were rpm (discrete), fish length (continuous) and their interaction. Since the fish for each scenario were not added all at once, but in samples of approximately 50 to 100 fish each time (not too many to avoid issues of overcrowding the pump and not too few to avoid poor statistical power), there might have been an unknown and unaccounted effect related to the time gap between samples. Therefore, the added value of providing the sample id as random effect in mixed logistic models was assessed by comparing the variation explained by the random effects with that explained by the fixed effects. For the different samples to be representative, each sample needed to contain at least 6 fish (some samples were much lower than anticipated due to the difference in the theoretical and observed scenario). Samples that contained less fish were not considered when developing the mixed models. Since, these mixed models provided singular fits, simple logistic models, rather than mixed logistic models, were used throughout and all samples, regardless of their number of fish, were used. To obtain the most parsimonious model, step-wise model selection with AIC (Akaike Information Criterion) as selection criteria was applied. For each scenario, 95% confidence intervals were estimated. In addition, predictor intervals were estimated using the aforementioned models for three length classes (with each length class containing approximately the same number of individuals). Since all eel samples and most control samples (exception for roach at 468 rpm with one severely injured fish exhibiting aberrant behavior) had a survival rate of 100%, they were not included as coefficients and standard errors would yield infinite values.

To compare the FNAFP and CAFP in terms of survival rate and injury, logistic and multinomial models were developed for roach with as explanatory variables the scenario (FNAFP at 468 rpm, FNAFP at 550 rpm and CAFP), fish length and their interaction. Model selection was done in the same way as for the previous models.

Since the type of injury determines to some extent the state (i.e. alive versus dead) and therefore the survival rate, it is to be expected that both are closely related. Nevertheless, separate analyses to assess the factors affecting the probability of a specific type of injury are still advised. When assessing injury, fish that were dead but had no external injury were omitted. Decision trees with as response the injury class (i.e. no injury, slight injury, severe injury) and injury (e.g. 2.1, 2.2, 2.3) and as explanatory variables the species (discrete), rpm (discrete) and fish length (continuous) were developed. Finally, parallel to the logistic models to assess the survival probability, multinomial models with as response the injury and injury class were developed. To obtain the most parsimonious model, step-wise model selection with AIC as selection criteria was applied. For each scenario, 95% confidence intervals were estimated.

### Passive sensor deployment and data analysis

#### Barotrauma detection system specifications and use

Each BDS sensor consists of a cylindrical 140 mm long, 40 mm diameter polycarbonate tube with two machined polyoxymethylene end caps, sensor electronics and is powered using two AAA alkaline batteries and has a total dry mass of 147 g. The sensors are neutrally buoyant and record the total water pressure at a rate of 100 Hz. The pressure sensors have a maximum pressure of 2000 hPa (MS5837-2BA, TE Connectivity, Switzerland) with a rated sensitivity of 0.02 hPa ($\sim$0.2 mm water column). In this study, pressure sensor data were recorded at a resolution of 0.01 hPa with 1.0 hPa ($\sim$10 mm water column) accuracy. Prior to sensor field deployment, the accuracy of each sensor was determined by laboratory testing in a custom-fabricated barochamber at total pressures up to 5500 hPa, which is a factor or 2.75 greater than the maximum rated pressure to reduce the risk of failure in the field. A commercial pressure sensor (HOBO U20-001-02, Onset Computer Corp., USA) was used to log pressure in the barochamber and to verify the accuracy of each BDS. The BDS is activated first in air with a magnetic switch, and the local atmospheric pressure is calculated as the mean value over 15 s. Afterwards, the device automatically sets each pressure sensor to a reference value of 1000 hPa. Neutral buoyancy of the sensors is achieved by screwing in or out the flat end cap, which increases or decreases the volume of the housing. Three identical digital sensors measure the total water pressure, which is the sum of the atmospheric, hydrostatic, and hydrodynamic pressures experienced during pump passage.

The sensor field tests were carried out over the course of three days from the 11th–13th of December, 2018. The field deployment consists of four stages. First, the sensors were turned on in air using the magnetic switch and the atmospheric pressure compensation was performed, this time was recorded as the sensor start time in the field protocol. Next, the sensors were injected into the pumping station by hand dropping them into the inlet region, and were shortly thereafter drawn into the pump without further intervention, this study did not use a forced injection system. After pump passage, the sensors were recovered with a net within 2–5 min after deployment. Over a total of 186 sensor deployments, five sensors were lost and ten datasets were unusable due to severe damage to the sensors (e.g. ruptured housing, crushed batteries). A total of 6 datasets from 13 sensor deployments were obtained for the axial flow pump, 64/80 from the FNAFP at 468 rpm and 51/63 from the FNAFP at 550 rpm. A total of 15/15 BDS deployments for each of the two control scenarios (468 and 550 rpm) were recovered and processed successfully. After net recovery, binary files from each sensor deployment were saved to a laptop for post-processing to evaluate the risk of barotrauma during pump station and control passage.

#### BDS data processing

The Regions of Interest (ROI) were manually identified for each pressure time series using the average pressure of the three BDS pressure sensors, unless explicitly mentioned otherwise. Outliers were defined as measurements with an average pressure of 10$^5$ hPa, and these pressure sensors datasets were removed before averaging the time series.

**Fig. 11:**
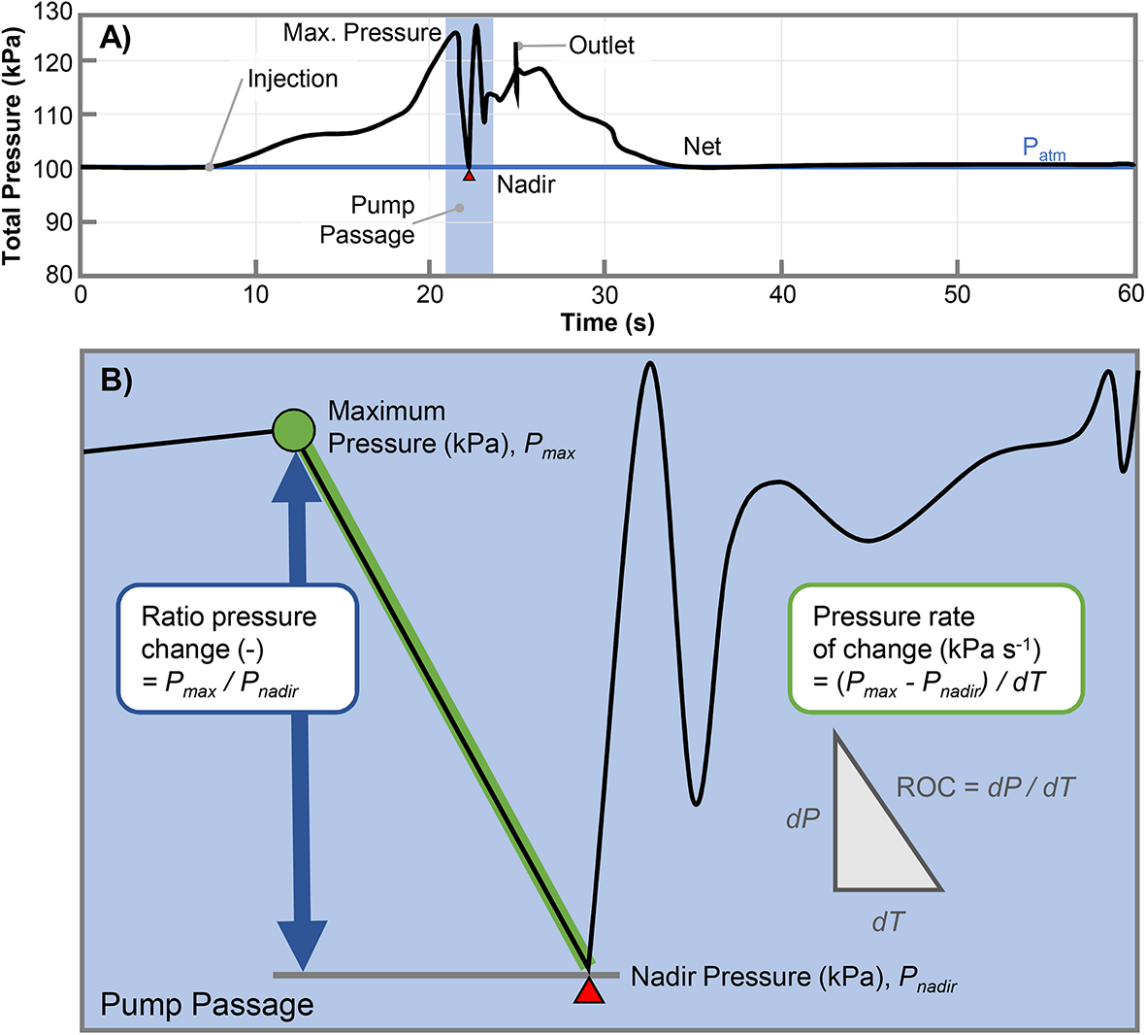
Schematic overview of pump passage pressure time series and the derivation of the three barotrauma parameters evaluated in this work. (**A**) Typical pressure time series during pump passage showing the injection, nadir pressure, and exit to the net. (**B**) Pressure parameters are evaluated based on the nadir pressure, ratio pressure change and maximal rate of pressure change, all of which occur in the immediate vicinity of the pump impeller.

The time of injection was taken as the first pressure reading exceeding a value of 1005 hPa (100.5 kPa). Determination of the nadir pressure was different for control and pump passage sensor data. The pump passage nadir was located between 3 and 75 s after the moment of injection (Fig. 11). As the pump passage nadir is characterized by a sudden decrease in the total pressure as it passes the impeller, the relative change in average pressure can be used to automatically identify the nadir value once the time of injection is known. A Savitzky–Golay smoothing filter was applied to the pressure time series data using a 2nd order polynomial a filter window size of 11 samples. The nadir was detected in time (< 0.1 s) based on the absolute change in the smoothed time series pressure using a threshold difference of 20  hPa. Finally, the lowest value found in the time series meeting these requirements was identified as the nadir. For the control samples, the nadir was located within 5 to 75 s after the time of injection. Unlike the pump passage data, there was no requirement to smooth the data for the control pressure time series, and the lowest pressure overall during control passage was taken as the nadir. After visual verification of all pressure sensor data, no manual corrections were needed.

#### Comparison of sensor data-derived barotrauma parameters

To assess whether there were any significant differences in the sensor-based barotrauma parameters between scenarios, Kruskal–Wallis tests were used. The null hypothesis is that there is no difference in the BDS ensemble mean pressure parameter (nadir, LRP and ROC) between pumps and scenarios. In case a significant effect was detected, pairwise Wilcoxon signed-rank sum tests with Bonferroni correction were used to compare among the scenarios. The nadir pressure is a key aspect for the assessment of barotrauma occurrence and simple averaging of the pressure through time over the sensors would have smoothed away the nadir pressure as it occurred at slightly different moments since injection for the different sensors. For visualization purposes it was therefore decided to depict the three most interesting moments in time (i.e. injection, nadir and exit) by defining them as references in a pressure-time plot. More specifically, to account for the variable duration between the moment of injection, nadir pressure and exit between sensors, the time series data were normalized to a passage duration interval from 0 (injection) to 1 (exit from the pump into the tailwater) with the center value (0.5) at the pressure nadir. The time normalization method uses linear registration to interpolate (using the average) each time series^46^. In this work, we used 500 equally spaced time points between the injection and nadir, and 500 equally spaced time points between the nadir and the exit. Plots of the average and median pressures as a function of the normalized passage duration were constructed.

#### Comparison of sensor data-derived strain rates

Based on Eq. (1) derived from Achenbach et al.^47^, BDS pressure data can be directly converted into strain rate estimates and compared to a threshold value. In the literature, 500 s$^{-1}$ has been recommended as a threshold value for shear stress-related injuries^37^ on juvenile rainbow trout (*Oncorhynchus mykiss*), spring and fall chinook salmon (*O. tshawytscha)*, and American shad (*Alosa sapidissima*). Therefore, this value was used as a threshold value to know when shear stress-related injuries could occur and for how long these events occurred.

1 $$\begin{aligned} \varepsilon \approx \frac{40 \Delta P}{\mu \sqrt{Re}} \end{aligned}$$

The pressure differentials between the central pressure sensor and the left or right pressure sensor, denoted as $\Delta$P_cl_ and $\Delta$P_cr_, were calculated for the entire pump passage. Using the obtained pressure differentials, the left ($\varepsilon$_cl_) and right strain rates ($\varepsilon$_cr_) were calculated using Eq. (1), after which the mean strain rate was calculated for the pump impeller passage. Given that the water temperature and the velocity are parameters influencing the Reynolds number, the water temperature recorded during the BDS deployments (4.5$\,^{\circ }\hbox {C}$), was used for the strain rate calculation. During this study no CFD modelling was performed and therefore pump-specific estimates of the velocity within the pump were not available. However, based on a previous work with a similar pump design, we assumed a range of possible velocities^48^ between 5 and 15 m s$^{-1}$. To determine the appropriate velocity for further calculations, the corresponding pressure differentials at a strain rate of 500 s$^{-1}$ were calculated using Eq. (1) for velocities of 5, 10, and 15 m s$^{-1}$. At a temperature of 4.5$\,^{\circ }\hbox {C}$, the resulting pressure differentials were 3.45 hPa, 4.86 hPa, and 5.95 hPa, respectively. After visually assessing the thresholds at these velocities, a velocity of 5 m s$^{-1}$ was selected. This choice was guided by the fact that at 5 m s$^{-1}$ the highest strain rates were produced and, following the precautionary principle, these values were deemed as the most suitable for estimating the possible onset of shear-related injuries. Furthermore, since the pressure differentials were significantly elevated when the threshold was exceeded, the differences between the thresholds at various velocities did not affect the occurrence of threshold exceedance. According to the literature, a strain rate of 500 s$^{-1}$ serves as a threshold for shear stress-related injuries^37^. This value was adopted as a benchmark to determine the potential onset of such injuries. The intersection between the 500 s$^{-1}$ threshold and the calculated mean strain rate was used to determine the duration for which the strain rate exceeded the injury threshold. The durations during which the strain rate exceeded 500 s$^{-1}$ were reported, along with the corresponding average shear stress values.

## Supplementary Information

Supplementary Information.

## Data Availability

The data (https://doi.org/10.5281/zenodo.14160723) and code (https://doi.org/10.5281/zenodo.14163421) to reproduce the results of this study have been made available via zenodo and github.
