# Supplementary material for: A fish-friendly axial flow pump turns out to be eel safe, roach unfriendly and bream unsafe
Source: Sci Rep. 2024 Dec 4;14:30234. doi: 10.1038/s41598-024-81095-6 (PMC11618692; doi:10.1038/s41598-024-81095-6)
Supplement: Supplementary file 1 — Supplementary Information. [file 41598_2024_81095_MOESM1_ESM.pdf]

Supplementary information to "A fish-friendly axial flow pump turns out to be eel safe, roach unfriendly and bream unsafe"

Stijn Bruneel[1,*], Ine S. Pauwels[1], Sarah Broos[1,2,3], Lore Vandamme[1], Jeroen Van Wichelen[1], Johan Coeck[1], Gert Toming[2], Jeffrey A. Tuhtan[2], and David Buysse[1]

[1]Research Institute for Nature and Forest (INBO), Havenlaan 88 bus 73, 1000 Brussels, Belgium
[2]Department of Computer Systems, Tallinn University of Technology
[3]Hydraulics Laboratory, Ghent University
[*]stijn.bruneel@inbo.be

ABSTRACT

Additional and refurbished pumping stations are required to mitigate the effects of climate change. These installations negatively impact threatened freshwater fish populations due to the increased risk of injury and mortality when fish pass through them. Fish friendly pumping installations have been proposed as a potential solution to reduce these risks. However, published assessments of these new types of pumps remains lacking, and the few available studies do not enable a cross-comparison with conventional pump types. The promising, yet understudied, Fairbank Nijhuis 'fish friendly' axial flow pump has been assessed in previous works, however the results remain ambiguous due to low recapture rates, unconsidered parameters, fixed operating conditions, and the inability to identify the likely sources of injury and mortality. In this study, we address the limitations of previous works by implementing a standardized protocol for live fish in conjunction with passive barotrauma detection sensors. The major finding of this work is that safe passage of eel (100% survival) is confirmed, but that bream and roach had a much lower survival probability (24% and 70% survival respectively) than expected, albeit higher than for a conventional axial flow pump (roach survival: 23%). Furthermore, roach and bream passing at higher rpm suffered significantly higher mortalities. The impact of the impeller was found to be the most common source of severe injury for all fish species in the axial flow pump, and for bream in the fish friendly pump at 550 rpm. These results are significant because they conclusively show that fish friendly pumps may be considered safe for eel, but not for other endemic European fish species such as roach and bream

1 Fish length

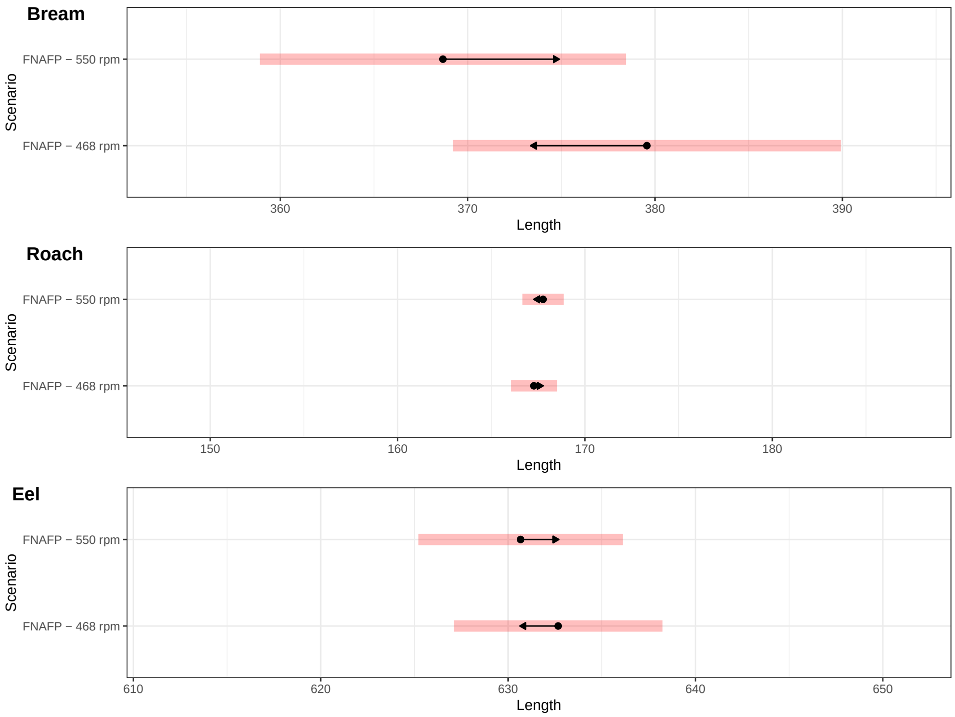

Figure Supplementary Fig. S1. Model output of the ANOVA fish length models for bream, roach and eel. 95 % confidence intervals are given per species and scenario. FNAFP = Fairbanks Nijhuis Axial Flow Pump.

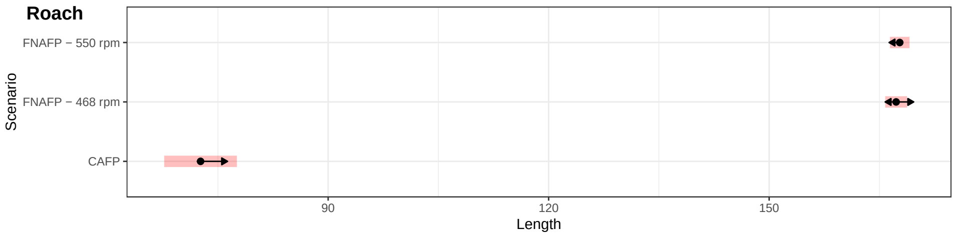

Figure Supplementary Fig. S2. Model output of the ANOVA fish length models for roach for which both the forced experiment and natural passage data was considered. 95 % confidence intervals are given per scenario. FNAFP = Fairbanks Nijhuis Axial Flow Pump; CAFP = Conventional Axial Flow Pump.

2 Fish survival

Table Supplementary Table S1. Model output of the most parsimonious logistic survival models for bream and roach. For each variable, the coefficient estimate (Est), standard error (SE), z-value, and p-value are given. FNAFP = Fairbanks Nijhuis Axial Flow Pump; CAFP = Conventional Axial Flow Pump.

Species	Factor	Est	SE	z-value	p-value
Bream	Intercept	5.735	2.356	2.434	0.015
	FNAFP - 550 rpm	-5.419	3.060	-1.771	0.077
	Length	-0.018	0.006	-2.787	0.005
	FNAFP - 550 rpm: Length	0.013	0.008	1.542	0.123
Roach	Intercept	5.907	1.943	3.041	0.002
	FNAFP - 550 rpm	-4.431	2.666	-1.662	0.096
	Length	-0.030	0.011	-2.584	0.010
	FNAFP - 550 rpm: Length	0.026	0.016	1.623	0.105

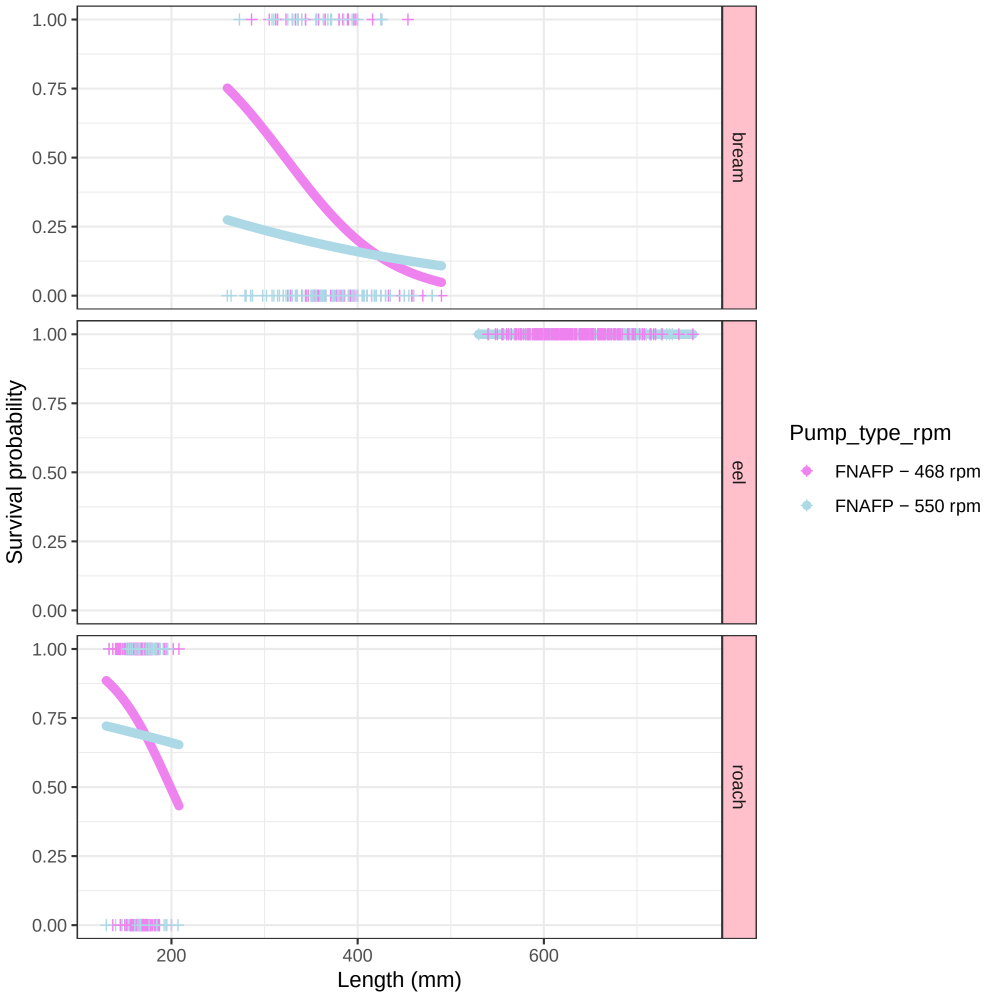

Figure Supplementary Fig. S3. Model output of the most parsimonious logistic survival models for bream, eel and roach. Predicted survival probability is depicted in function of fish length (lines) per species and scenario (rpm). The original data is presented as crosses. FNAFP = Fairbanks Nijhuis Axial Flow Pump; CAFP = Conventional Axial Flow Pump.

Table Supplementary Table S2. Model output of the most parsimonious logistic survival model for roach for which both the forced experiment and natural passage data was considered. For each variable, the coefficient estimate (Est), standard error (SE), z-value, and p-value are given. FNAFP = Fairbanks Nijhuis Axial Flow Pump; CAFP = Conventional Axial Flow Pump.

Factor	Est	SE	z-value	p-value
Intercept	1.091	0.607	1.798	0.072
FNAFP - 468 rpm	2.855	0.790	3.614	0.000
FNAFP - 550 rpm	2.742	0.789	3.474	0.001
Length	-0.018	0.007	-2.601	0.009

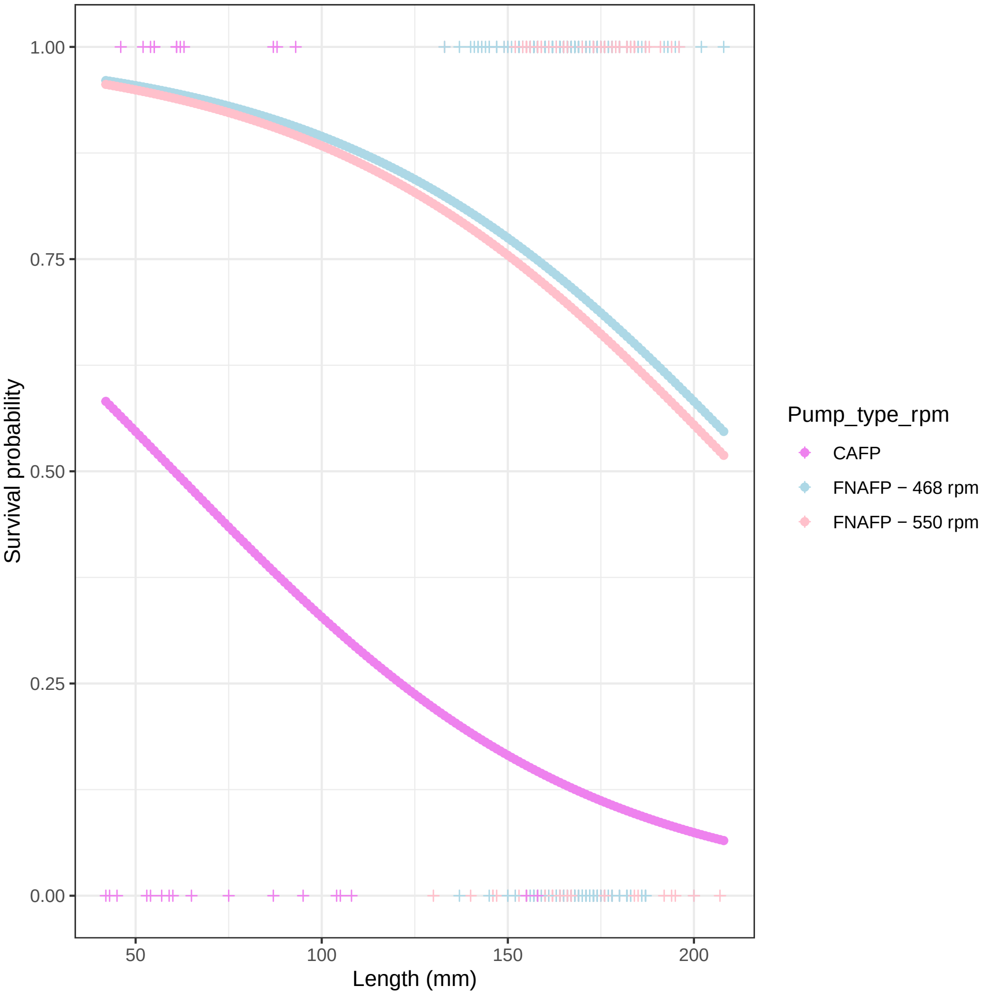

Figure Supplementary Fig. S4. Model output of the most parsimonious logistic survival models for roach for which both the forced experiment and natural passage data was considered. Predicted survival probability is depicted in function of fish length (lines) per scenario. The original data is presented as crosses. FNAFP = Fairbanks Nijhuis Axial Flow Pump; CAFP = Conventional Axial Flow Pump.

3 Fish injury

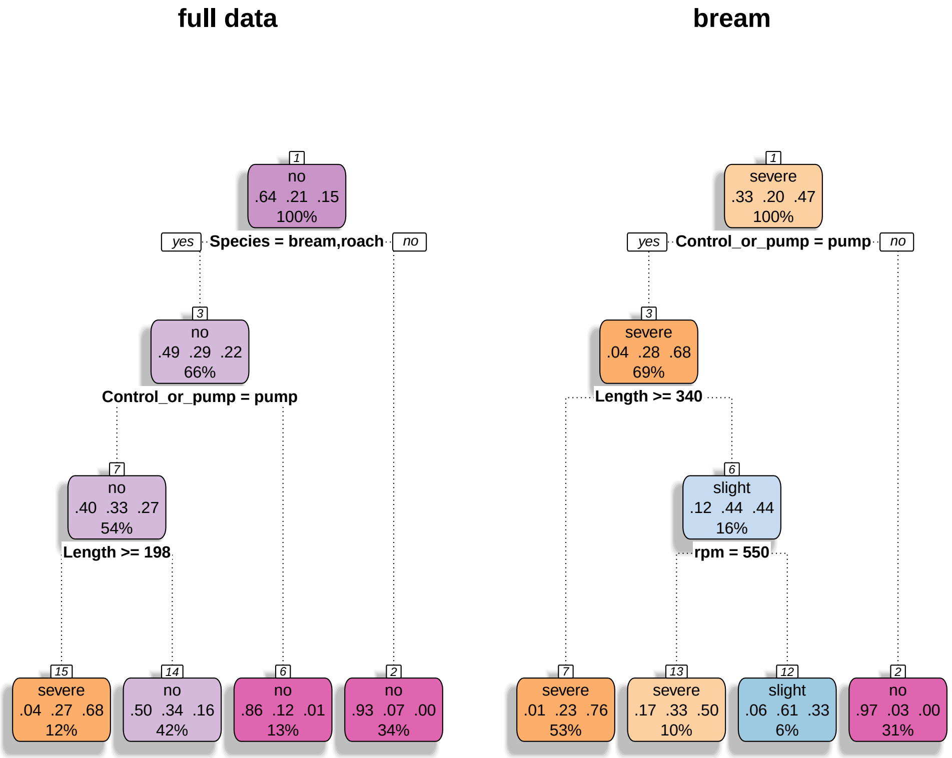

Figure Supplementary Fig. S5. Decision trees with as multinomial response the injury class (no, slight or severe). For the decision tree constructed with all data of the FNAFP (Fairbanks Nijhuis Axial Flow Pump), the factors species, type, rpm and fish length were provided. For the decision tree constructed with the bream data, the factors type, rpm and fish length were provided. The first three numbers next to each other in each branch give the proportion of no (left) , slight (middle) and severe (right) injuries. The fourth number at the bottom gives the percentage of fish the node applies to. Nodes are labeled as no, slight or severe when the proportion of dead fish is higher or lower than 33.33 % respectively. Nodes are numbered in the following way: the main branch at the top receives the number one and from there on, the numbers increase from left-to-right and downwards. The first branch under the main branch to the left will receive number two and the second branch under the main branch to the right will receive the number three, and so on. Some numbers are skipped because non-existing nodes are also numbered. Left branches always represent an affirmation to the question at each parting of branches. The color range from pink to blue indicate whether fish were more likely to be injured or not.

Table Supplementary Table S3. Model output of the most parsimonious multinomial injury models for the FNAFP (Fairbanks Nijhuis Axial Flow Pump) for bream and roach. For each variable, the coefficient estimates are given. Significant estimates ($p < 0.05$) were given an asterisk.

Species	Injury	Intercept	FNAFP - 550 rpm	Length
Bream	slight	-3.455*	-1.101	0.0176*
	severe	-7.144*	-0.406	0.0291*
Roach	slight	-0.976	-0.273	0.00442
	severe	-6.945*	0.215	0.0339*

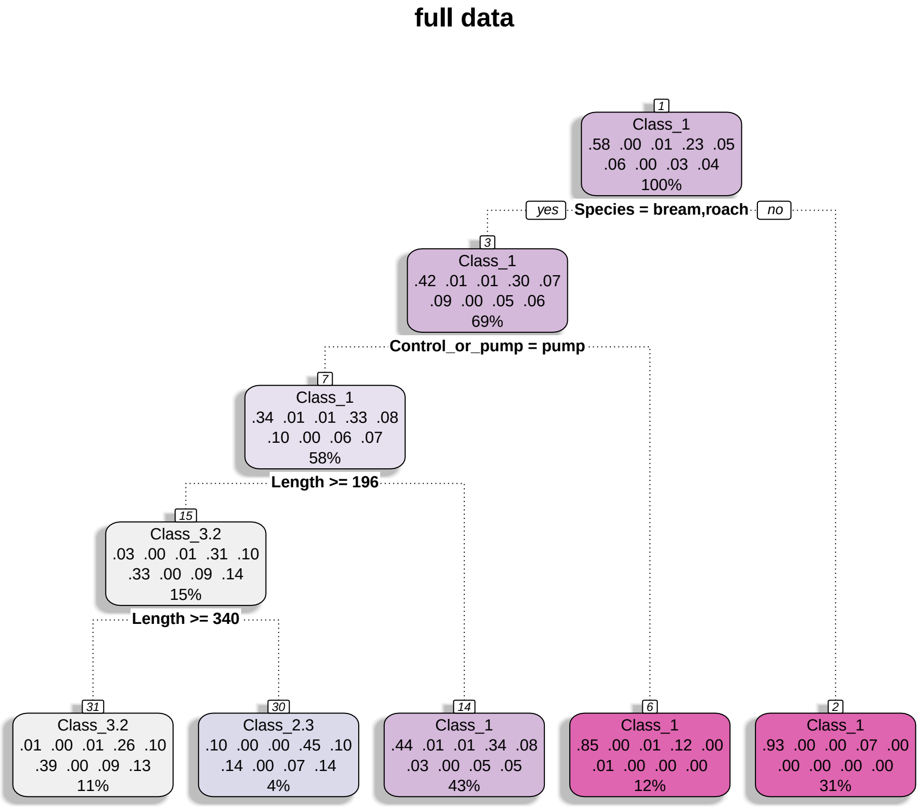

Figure Supplementary Fig. S6. Decision tree for all the data of the FNAFP (Fairbanks Nijhuis Axial Flow Pump) with as multinomial response the injury. For the decision tree the factors species, type, rpm and fish length were provided. For the decision tree constructed with the bream data, the factors type, rpm and fish length were provided. The first nine numbers next to each other in each branch give the proportion of the different types of injuries. The last number at the bottom gives the percentage of fish the node applies to. Nodes are labeled as one of the different types of injury when the proportion of dead fish is higher or lower than 11.11 % respectively. Nodes are numbered in the following way: the main branch at the top receives the number one and from there on, the numbers increase from left-to-right and downwards. The first branch under the main branch to the left will receive number two and the second branch under the main branch to the right will receive the number three, and so on. Some numbers are skipped because non-existing nodes are also numbered. Left branches always represent an affirmation to the question at each parting of branches. The color range from pink to blue indicate whether fish were more likely to be injured or not.

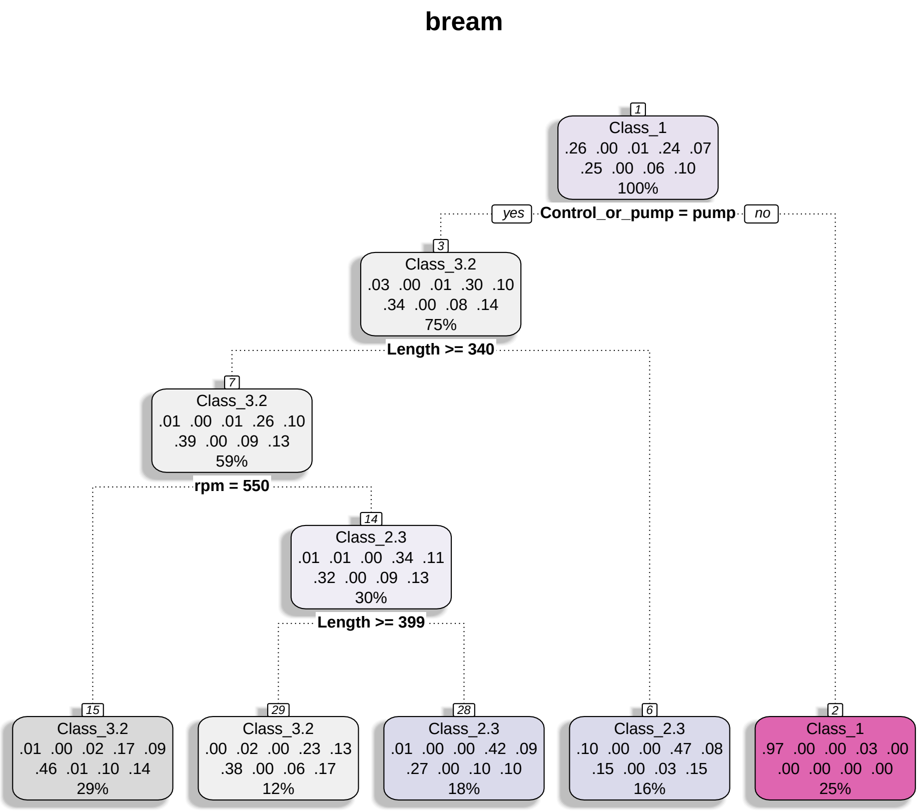

Figure Supplementary Fig. S7. Decision tree for bream for the FNAFP (Fairbanks Nijhuis Axial Flow Pump) with as multinomial response the injury. For the decision tree the factors type, rpm and fish length were provided. The first nine numbers next to each other in each branch give the proportion of the different types of injuries. The last number at the bottom gives the percentage of fish the node applies to. Nodes are labeled as one of the different types of injury when the proportion of dead fish is higher or lower than 11.11 % respectively. Nodes are numbered in the following way: the main branch at the top receives the number one and from there on, the numbers increase from left-to-right and downwards. The first branch under the main branch to the left will receive number two and the second branch under the main branch to the right will receive the number three, and so on. Some numbers are skipped because non-existing nodes are also numbered. Left branches always represent an affirmation to the question at each parting of branches. The color range from pink to blue indicate whether fish were more likely to be injured or not.

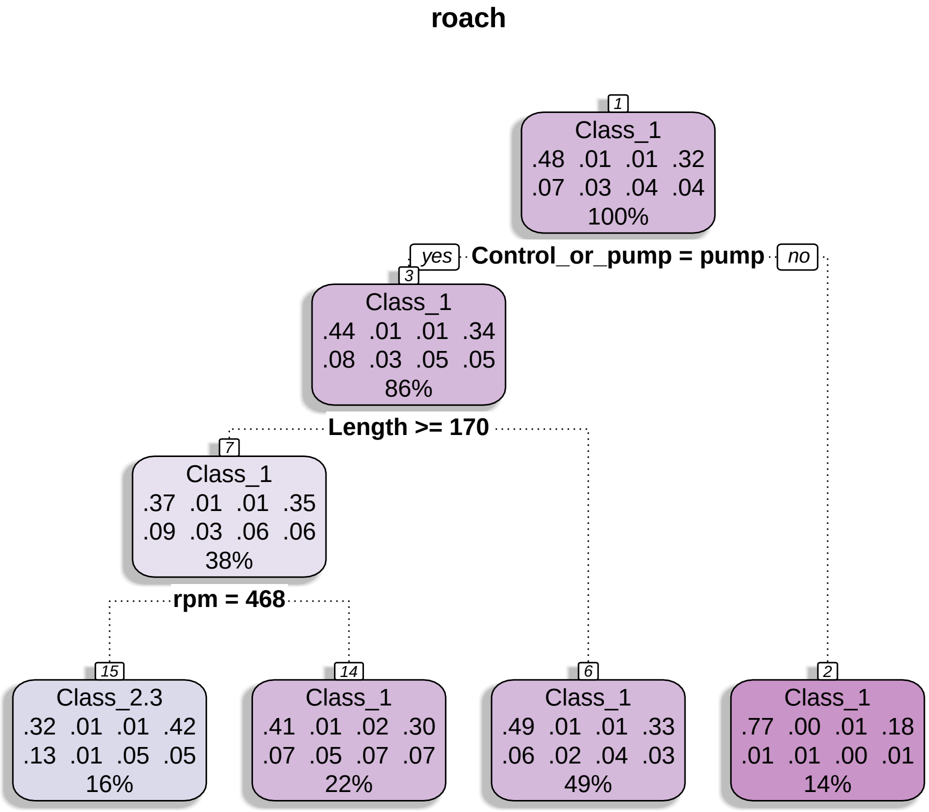

Figure Supplementary Fig. S8. Decision tree for roach for the FNAFP (Fairbanks Nijhuis Axial Flow Pump) with as multinomial response the injury. For the decision tree the factors type, rpm and fish length were provided. The first eight numbers next to each other in each branch give the proportion of the different types of injuries. The last number at the bottom gives the percentage of fish the node applies to. Nodes are labeled as one of the different types of injury when the proportion of dead fish is higher or lower than 12.50 % respectively. Nodes are numbered in the following way: the main branch at the top receives the number one and from there on, the numbers increase from left-to-right and downwards. The first branch under the main branch to the left will receive number two and the second branch under the main branch to the right will receive the number three, and so on. Some numbers are skipped because non-existing nodes are also numbered. Left branches always represent an affirmation to the question at each parting of branches. The color range from pink to blue indicate whether fish were more likely to be injured or not.

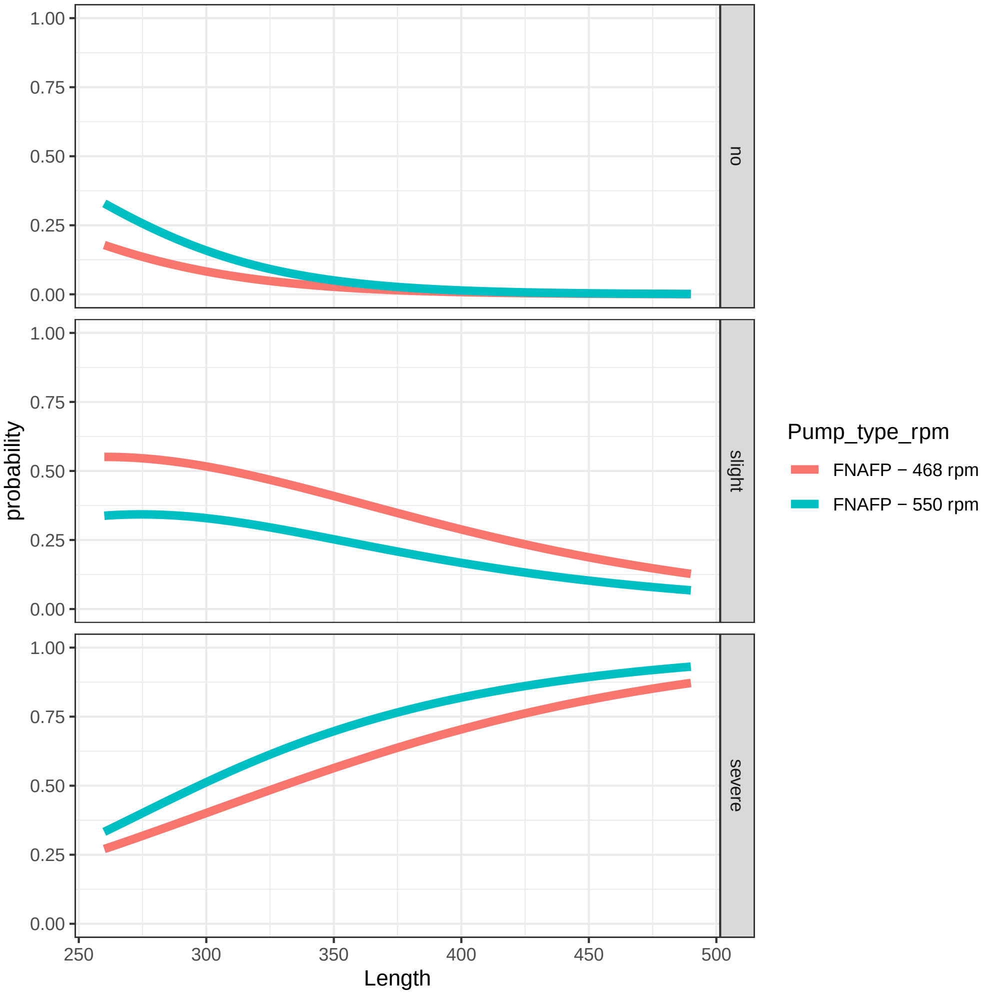

Figure Supplementary Fig. S9. Model output of the most parsimonious multinomial injury model for the FNAFP (Fairbanks Nijhuis Axial Flow Pump) for bream. Predicted injury probability is depicted in function of fish length (lines) per injury class and scenario (rpm).

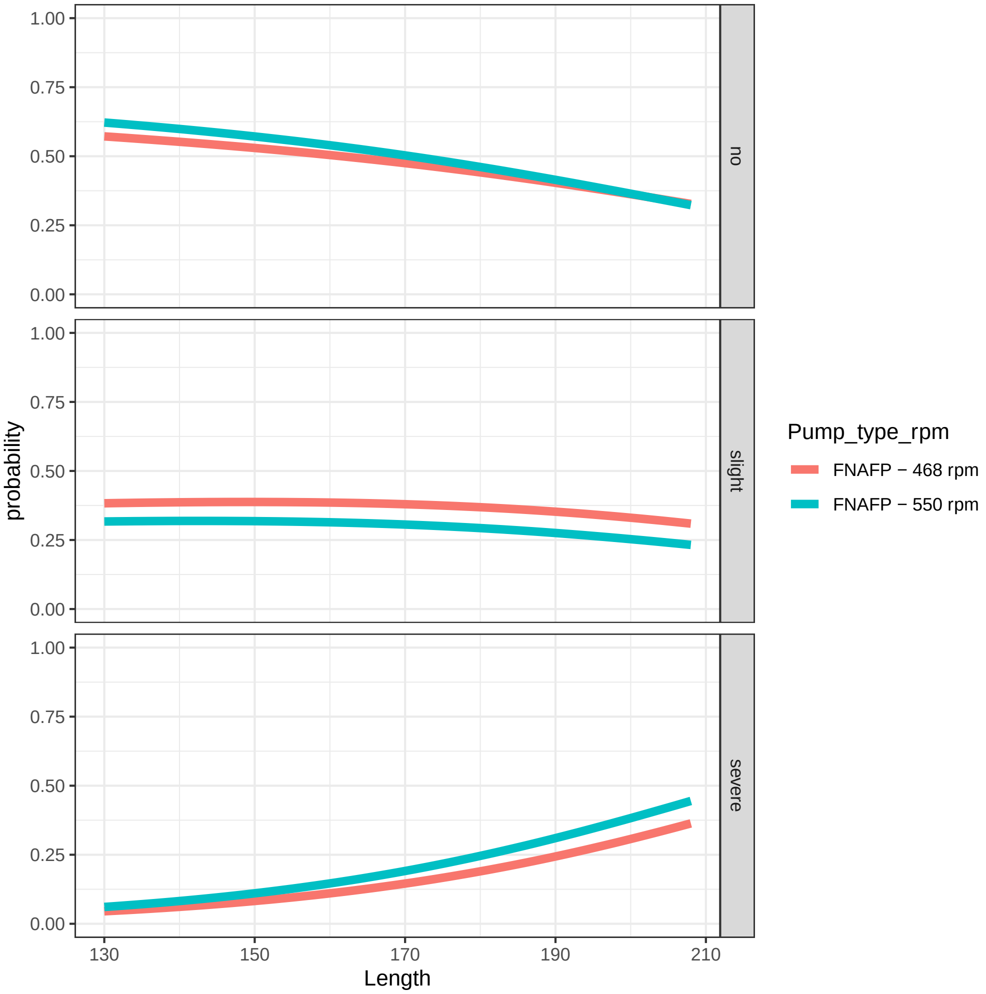

Figure Supplementary Fig. S10. Model output of the most parsimonious multinomial injury model for the FNAFP (Fairbanks Nijhuis Axial Flow Pump) for roach. Predicted injury probability is depicted in function of fish length (lines) per injury class and scenario (rpm).

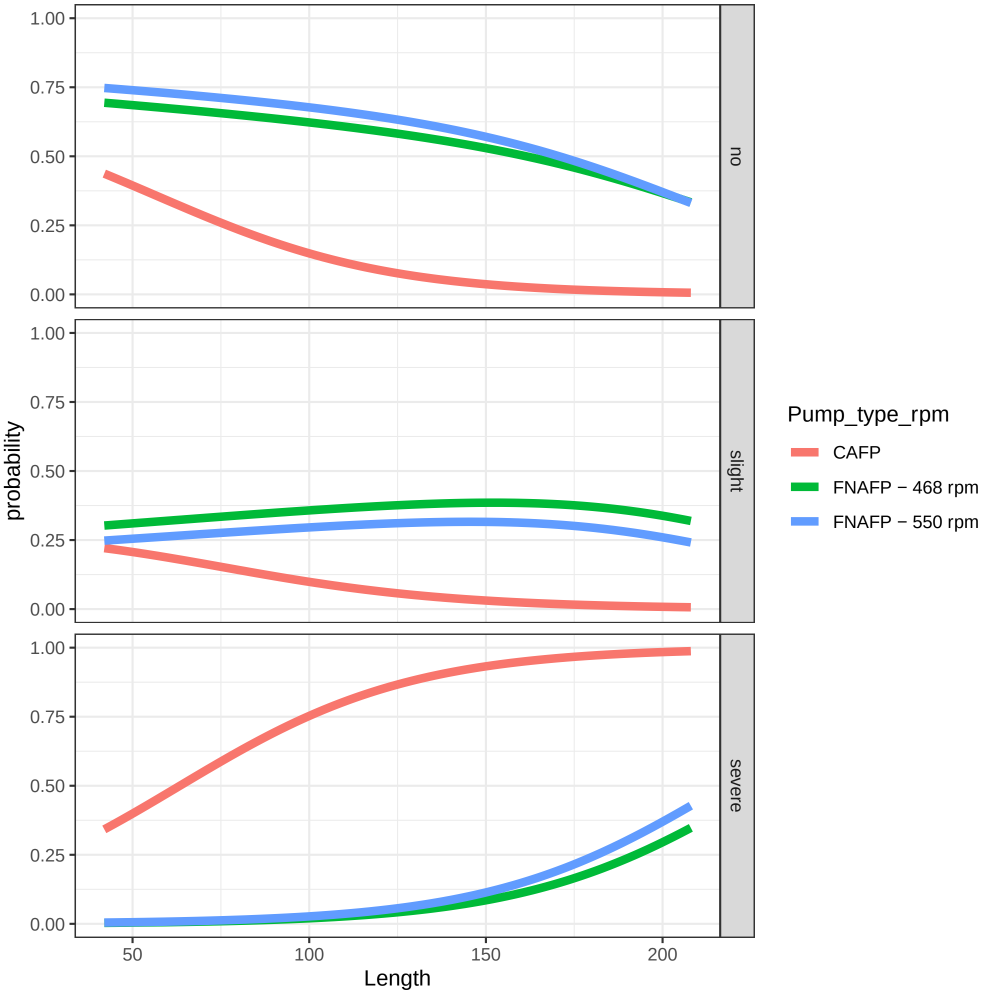

Figure Supplementary Fig. S11. Model output of the most parsimonious multinomial injury model for roach for which both the forced experiment (FNAFP) and natural passage (CAFP) data was considered. Predicted injury probability is depicted in function of fish length (lines) per injury class and scenario (rpm). FNAFP = Fairbanks Nijhuis Axial Flow Pump; CAFP = Conventional Axial Flow Pump.

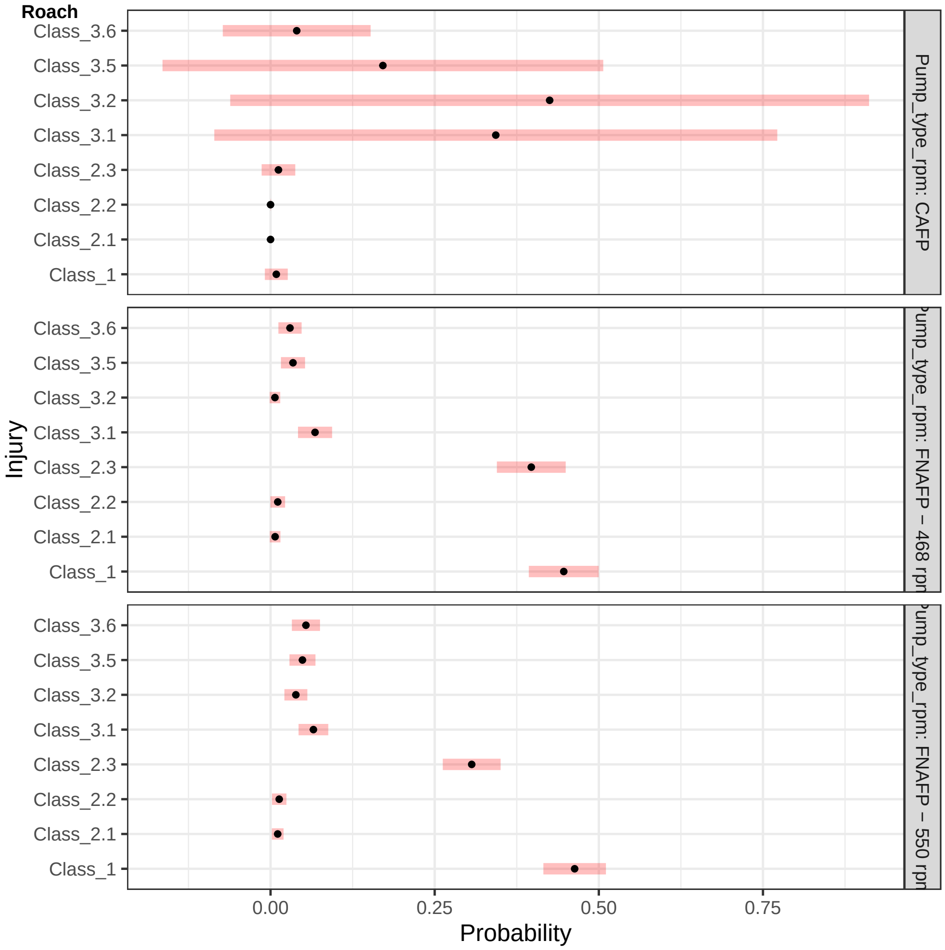

Figure Supplementary Fig. S12. Model output of the most parsimonious multinomial injury models for roach of which both the forced experiment (FNAFP) and natural passage (CAFP) data was considered. 95 % confidence intervals are given per species and scenario. FNAFP = Fairbanks Nijhuis Axial Flow Pump; CAFP = Conventional Axial Flow Pump.

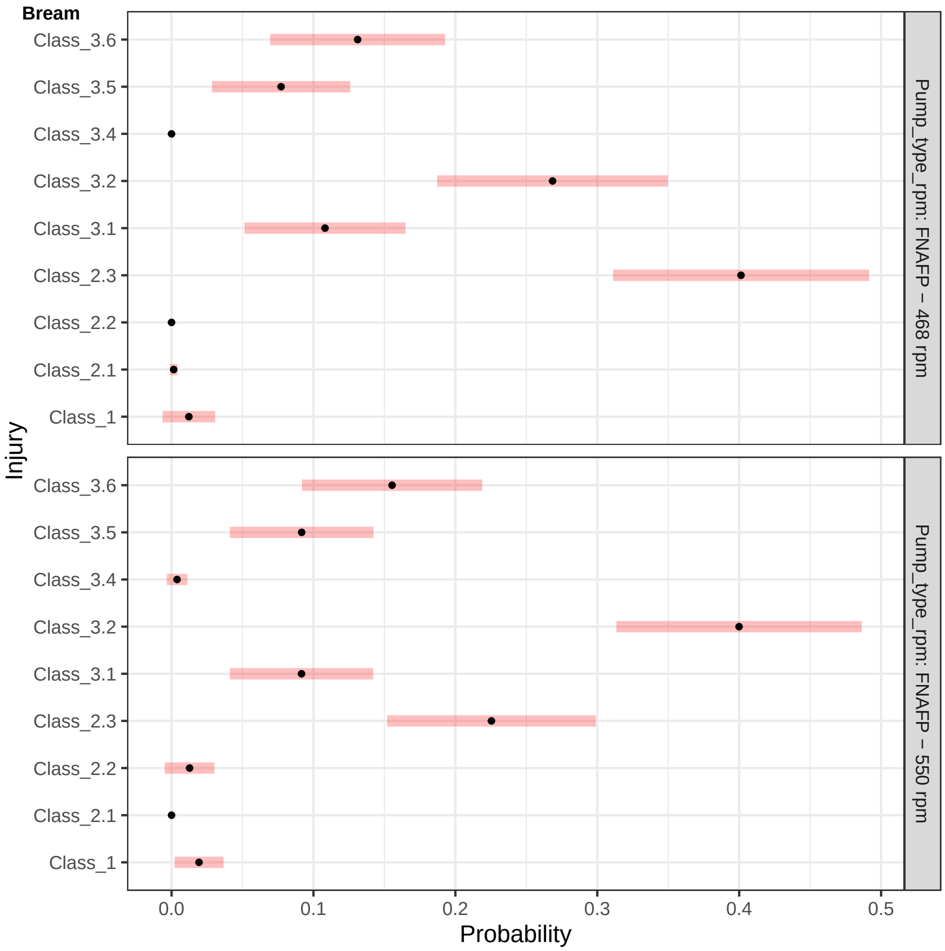

Figure Supplementary Fig. S13. Model output of the most parsimonious multinomial injury models for the FNAFP (Fairbanks Nijhuis Axial Flow Pump) for bream. 95 % confidence intervals are given per species and scenario.

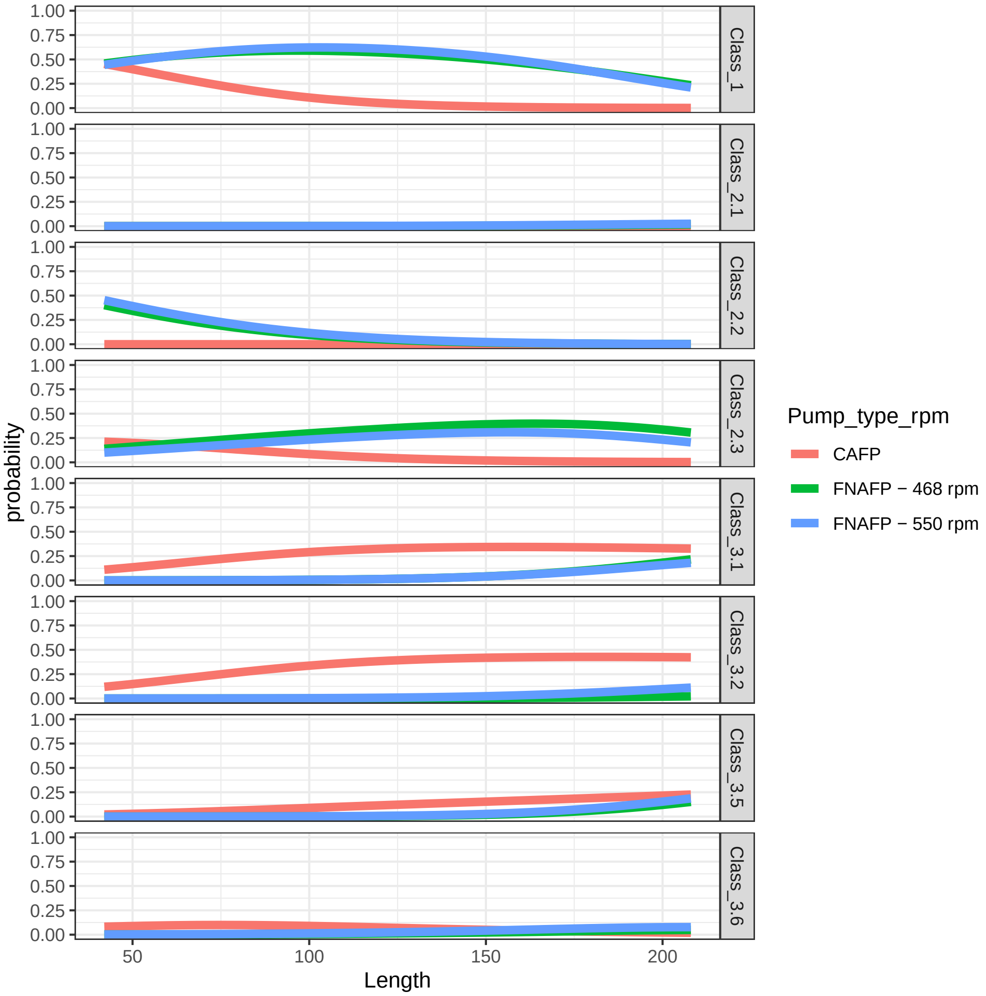

Figure Supplementary Fig. S14. Model output of the most parsimonious multinomial injury model for roach. Predicted injury probability is depicted in function of fish length (lines) per injury class and scenario (rpm). FNAFP = Fairbanks Nijhuis Axial Flow Pump; CAFP = Conventional Axial Flow Pump.

4 BDS

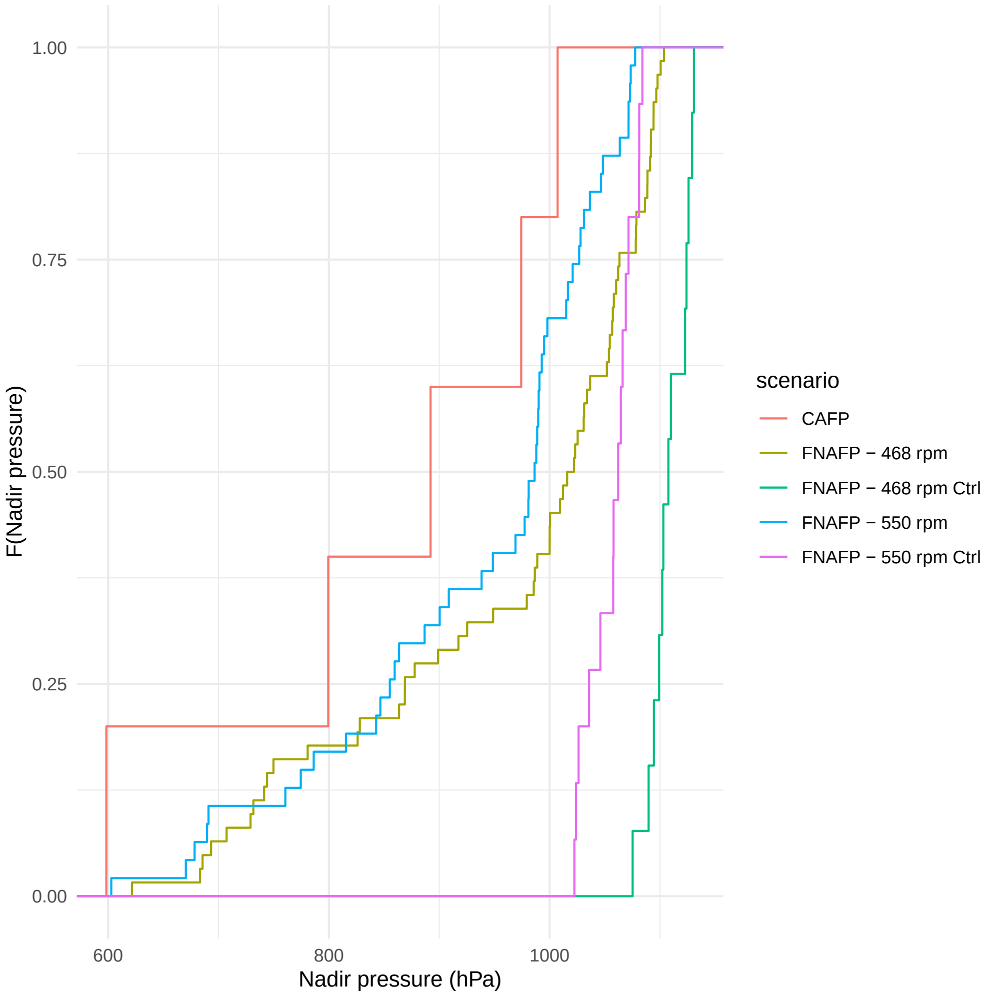

Figure Supplementary Fig. S15. Nadir pressure cumulative distribution functions (F) for the different scenarios. FNAFP = Fairbanks Nijhuis Axial Flow Pump; CAFP = Conventional Axial Flow Pump.

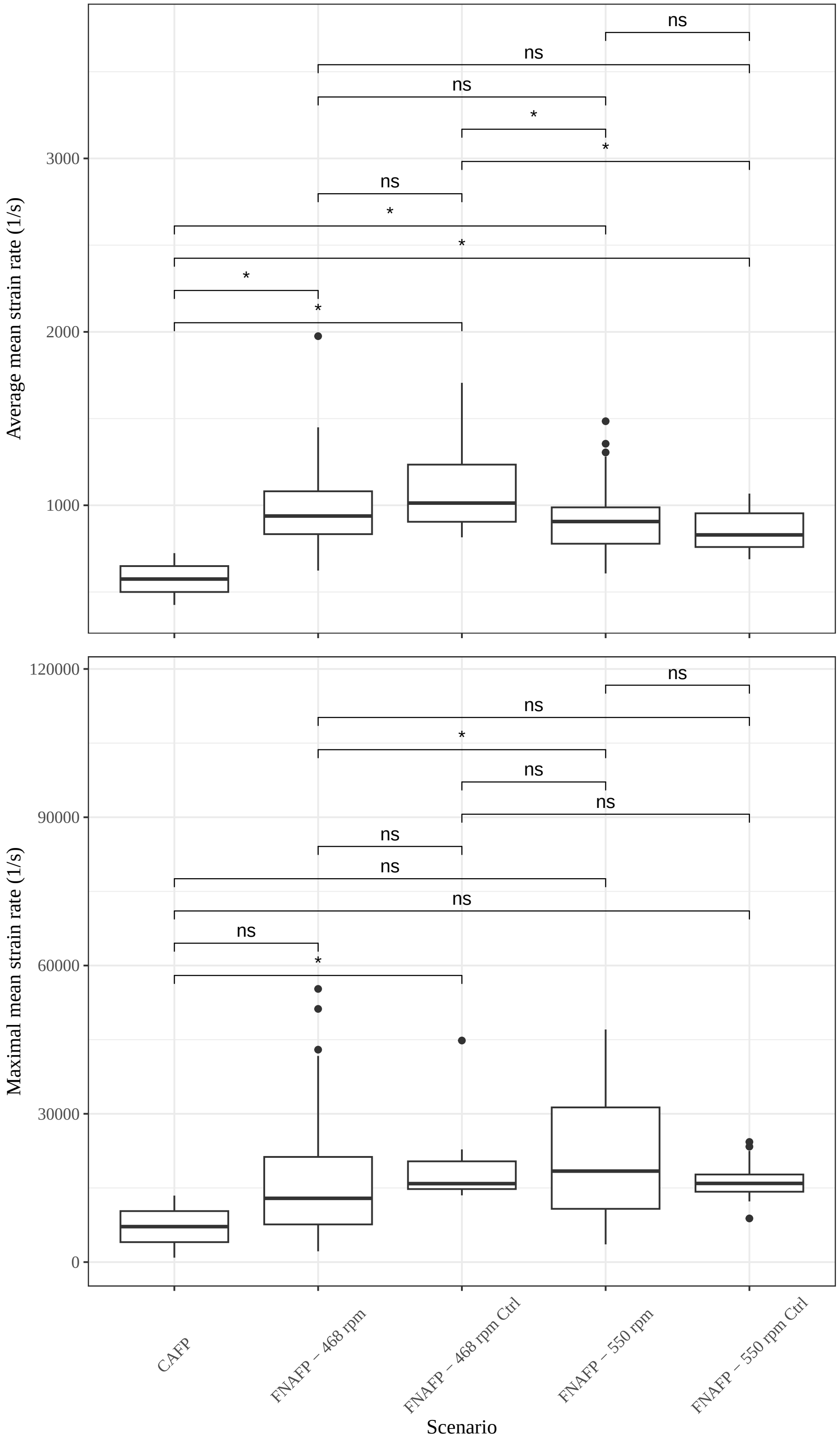

Figure Supplementary Fig. S16. Boxplots representing the average and maximal mean strain rate per pump type and operating scenario. Pairwise wilcoxon ranks sum tests with Bonferroni correction were applied. p-values were classified as non-significant or ns (>0.05), * (0.05-0.01), ** (0.01-0.001), *** (0.001-0.0001), **** (0.0001-0). FNAFP = Fairbanks Nijhuis Axial Flow Pump; CAFP = Conventional Axial Flow Pump; Ctrl = Control.

5 Power analyses

5.1 Prospective power analyses

Prior to data collection, the required number of fish for the FNAFP experiments (without consideration of control samples) was determined using Wilson score intervals. In order for the width of the 95% confidence interval to be no more than 20%, 100 fish per scenario would have been required.

5.2 Retrospective power analyses

Despite the flawed nature of retrospective (or post-hoc) power analyses to determine the sample size that would have been required to estimate the model coefficients with sufficient reliability, a careful evaluation of the number of individuals that would have been required to obtain significant values is still valuable. Especially as a baseline for future studies. The most parsimonious logistic survival model per species, developed for the full data, was run for different number of individuals. For each unique number of individuals, 10^4 random subsets of data were created and used to fit the model. The statistical power of each variable in the model was determined for every unique number of individuals.

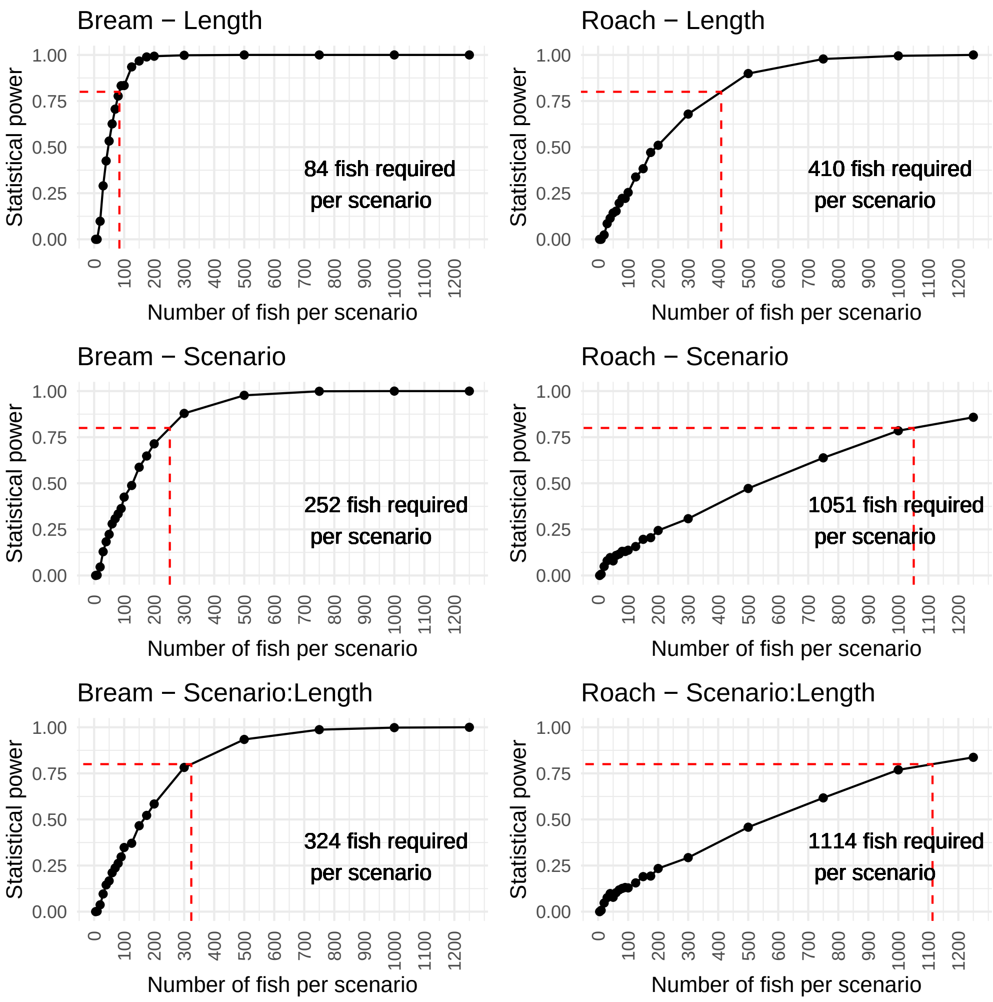

Figure Supplementary Fig. S17. Retrospective power analyses for the constructed logistic survival models for the FNAFP (Fairbanks Nijhuis Axial Flow Pump). For each species one logistic model was constructed. For both roach and bream, the factors length, scenario and their interaction were retained. The dotted red lines depict the required number of fish per scenario to obtain a statistical power of 0.8.
